# The PRK/Rubisco shunt strongly influences Arabidopsis seed metabolism and oil accumulation, affecting more than carbon recycling

**DOI:** 10.1093/plcell/koac338

**Published:** 2022-12-01

**Authors:** Gabriel Deslandes-Hérold, Martina Zanella, Erik Solhaug, Michaela Fischer-Stettler, Mayank Sharma, Léo Buergy, Cornelia Herrfurth, Maite Colinas, Ivo Feussner, Melanie R Abt, Samuel C Zeeman

**Affiliations:** Department of Biology, Institute of Molecular Plant Biology, ETH Zurich, CH-8092 Zurich, Switzerland; Department of Biology, Institute of Molecular Plant Biology, ETH Zurich, CH-8092 Zurich, Switzerland; Department of Biology, Institute of Molecular Plant Biology, ETH Zurich, CH-8092 Zurich, Switzerland; Department of Biology, Institute of Molecular Plant Biology, ETH Zurich, CH-8092 Zurich, Switzerland; Department of Biology, Institute of Molecular Plant Biology, ETH Zurich, CH-8092 Zurich, Switzerland; Department of Biology, Institute of Molecular Plant Biology, ETH Zurich, CH-8092 Zurich, Switzerland; Department for Plant Biochemistry, Albrecht von Haller Institute for Plant Sciences and Göttingen Center for Molecular Biosciences (GZMB), University of Göttingen, D-37077 Göttingen, Germany; Service Unit for Metabolomics and Lipidomics, Göttingen Center for Molecular Biosciences (GZMB), University of Göttingen, D-37077 Göttingen, Germany; Department of Biology, Institute of Molecular Plant Biology, ETH Zurich, CH-8092 Zurich, Switzerland; Department for Plant Biochemistry, Albrecht von Haller Institute for Plant Sciences and Göttingen Center for Molecular Biosciences (GZMB), University of Göttingen, D-37077 Göttingen, Germany; Service Unit for Metabolomics and Lipidomics, Göttingen Center for Molecular Biosciences (GZMB), University of Göttingen, D-37077 Göttingen, Germany; Department of Biology, Institute of Molecular Plant Biology, ETH Zurich, CH-8092 Zurich, Switzerland; Department of Biology, Institute of Molecular Plant Biology, ETH Zurich, CH-8092 Zurich, Switzerland

## Abstract

The carbon efficiency of storage lipid biosynthesis from imported sucrose in green Brassicaceae seeds is proposed to be enhanced by the PRK/Rubisco shunt, in which ribulose 1,5-bisphosphate carboxylase/oxygenase (Rubisco) acts outside the context of the Calvin–Benson–Bassham cycle to recycle CO_2_ molecules released during fatty acid synthesis. This pathway utilizes metabolites generated by the nonoxidative steps of the pentose phosphate pathway. Photosynthesis provides energy for reactions such as the phosphorylation of ribulose 5-phosphate by phosphoribulokinase (PRK). Here, we show that loss of PRK in *Arabidopsis thaliana* (Arabidopsis) blocks photoautotrophic growth and is seedling-lethal. However, seeds containing *prk* embryos develop normally, allowing us to use genetics to assess the importance of the PRK/Rubisco shunt. Compared with nonmutant siblings, *prk* embryos produce one-third less lipids—a greater reduction than expected from simply blocking the proposed PRK/Rubisco shunt. However, developing *prk* seeds are also chlorotic and have elevated starch contents compared with their siblings, indicative of secondary effects. Overexpressing PRK did not increase embryo lipid content, but metabolite profiling suggested that Rubisco activity becomes limiting. Overall, our findings show that the PRK/Rubisco shunt is tightly integrated into the carbon metabolism of green Arabidopsis seeds, and that its manipulation affects seed glycolysis, starch metabolism, and photosynthesis.

## Teaser

A genetic approach demonstrates surprisingly intimate metabolic integration of a pathway increasing carbon storage efficiency for oil biosynthesis in green Arabidopsis seeds.

**IN A NUTSHELL**

**Background:** Light promotes the accumulation of storage lipids during development of oilseeds with green embryos. This has been explained by embryonic photosynthesis generating cofactors that can power an energy-consuming metabolic pathway known as the PRK/Rubisco shunt that is distinct from the Calvin cycle operating in leaves. There is good biochemical evidence for the existence of this pathway in Brassicaceae; however, the extent of its biological significance has not been assessed genetically. Here, we use a refined genetic complementation approach in Arabidopsis to study the role of PRK, specifically in the proposed pathway in its green seeds.

**Questions:** How can we study the PRK/Rubisco shunt genetically, and what is its quantitative influence on storage oil accumulation in green, developing Arabidopsis seeds?

**Findings:** As an enzyme integral to the Calvin cycle, the complete loss of PRK is detrimental to plant growth. However, because heterozygous *PRK/prk* plants are phenotypically normal, we used them to establish a plant line generating *prk* embryos in parallel with complemented siblings, which can be differentiated using a fluorescent marker. The absence of PRK throughout embryogenesis reduced the oil content in the embryo by one-third; more than expected from theoretical calculations of the contribution of the PRK/Rubisco shunt. Several lines of evidence further indicate tight metabolic integration of the shunt into green embryo photosynthesis and metabolism.

**Next steps:** Our observations provide insight into the integration of the PRK/Rubisco shunt into Arabidopsis embryo metabolism. We would like to understand better how it is coordinated with pathways leading to other storage compounds, how it is regulated genetically and biochemically, and how this knowledge can help oil crop improvement.

## Introduction

Developing plant seeds rely on maternally derived substrates to sustain their metabolism and synthesize the storage compounds that fuel germination and seedling establishment. The cleavage of sucrose obtained from the mother plant yields hexose phosphates that supply metabolic processes such as starch biosynthesis and the respiratory pathways, i.e. glycolysis, the tricarboxylic acid (TCA) cycle, and the oxidative pentose phosphate pathway (OPPP), pathways that generate precursors for amino acid and lipid biosynthesis (Baud et al., 2008). Partitioning among these different pathways during seed development is dynamic and species dependent, resulting in some seeds accumulating starch as the predominant final storage compound (as in many grasses) and others accumulating lipids (as in many Brassicaceae).

The Brassicaceae include many commercially important oilseed species (e.g. *Brassica napus, Brassica rapa*, and *Brassica juncea*) and the widely studied model species *Arabidopsis thaliana* (Arabidopsis). Although the seeds of such species are oil-rich at maturity, they also transiently accumulate starch during development, prior to lipid accumulation (da Silva et al., 1997; Focks and Benning, 1998; Baud et al., 2002). The shift in the utilization of carbon resources during seed filling has been proposed to be the result of several developmental regulators, notably the transcription factor WRINKLED1 (WRI1) (Focks and Benning, 1998; Cernac and Benning, 2004).

Both starch and lipid biosynthesis draw on the hexose phosphate pool in the seed, but they differ significantly with regard to complexity, energy demand, carbon efficiency, as well as the final energy density attained. Starch biosynthesis happens exclusively inside plastids and utilizes ADP-Glucose (ADPGlc), a nucleotide-activated sugar derived directly from the interconversion of hexose phosphates, as substrate (see Pfister and Zeeman, 2016, for a comprehensive review). The numerous steps required for storage lipid biosynthesis are distributed among several cellular locations. Broadly speaking, storage lipid biosynthesis can be divided into three sequential parts: synthesis of the substrate pyruvate by glycolysis, fatty acid (FA) biosynthesis, and triacylglycerol (TAG) assembly.

Both the cytosol and the plastids contain the enzymes to perform glycolysis (Kang and Rawsthorne, 1994). Current evidence from *B. napus* and Arabidopsis suggests that, during seed filling, both the cytosol and plastids may be involved. Cytosolic glycolysis produces phosphoenolpyruvate (PEP) and, to some extent, pyruvate (Pyr), both of which can be imported to fuel the plastidial biogenesis of acetyl-CoA (Eastmond and Rawsthorne, 2000; White et al., 2000; Ruuska et al., 2002; Schwender et al., 2015). Acetyl-CoA is subsequently used by the plastidial acetyl-CoA carboxylase (ACCase), a multisubunit enzyme, to generate malonyl-CoA in a first committed step toward FA biosynthesis (Konishi et al., 1996; Thelen and Ohlrogge, 2002). Several sequential enzymatic activities then generate FAs, the majority of which are exported from the plastid. In the endoplasmic reticulum (ER), these exported FAs are transferred onto glycerol-3-phosphate (Gly-3P) to form TAGs, the final lipid storage form (see Baud et al., 2008, and references therein).

Lipid biosynthesis requires large amounts of energy (as ATP) and reducing power (as NADH, NADPH), and also releases one molecule of CO_2_ at the oxidative decarboxylation of Pyr to acetyl-CoA, catalyzed by pyruvate dehydrogenase (PDH). This reduces the carbon conversion efficiency by one-third with respect to starch biosynthesis (Baud and Lepiniec, 2010). Through elegant labeling experiments, Schwender and colleagues demonstrated that in developing Brassicaceae seeds, the Calvin–Benson–Bassham cycle (CBBC) enzymes phosphoribulokinase (PRK) and Rubisco act in a noncanonical metabolic context to recycle the CO_2_ released by PDH (Schwender et al., 2004). This PRK/Rubisco shunt utilizes substrates generated by the nonoxidative steps of the PPP, and the carboxylation action of Rubisco generates additional 3-phosphoglycerate (3PGA) while bypassing parts of glycolysis. The increased costs of this pathway, in terms of energy and reductant, are met by photosynthesis in the developing green seeds using the residual light transmitted through the surrounding seed coat and pod wall.

This proposed pathway is consistent with earlier findings showing that the photosynthetic electron transport chain is functional in isolated *B. napus* embryos (Asokanthan et al., 1997) and that seed lipid content is positively influenced by light in both *B. napus* and Arabidopsis (Ruuska et al., 2004; Li et al., 2006). Schwender et al. (2004) calculated that, in *B. napus*, Rubisco is responsible for up to half of the 3PGA molecules subsequently used for FA biosynthesis. This corresponds to an increased carbon efficiency of 10% compared with using glycolysis as the only source of 3PGA. This value is consistent with the increased carbon conversion efficiency of green *B. napus* seeds compared with nongreen sunflower seeds. Interestingly, in later studies, the contribution of Rubisco in the context of developing, green seeds was shown to vary significantly not only between species (25% and 90% of total plastidic 3PGA produced by Rubisco in pennycress and Arabidopsis, respectively (Lonien and Schwender, 2009; Tsogtbaatar et al., 2020)) but also between different genotypes of the same species (*B. napus:* between 42% and 68% of 3PGA molecules originating from Rubisco activity in different entries; Schwender et al., 2015).

The above-mentioned discoveries have provided initial insight into the relevance of Rubisco-mediated carbon fixation in the context of green-seed metabolism. Here, we provide genetic evidence for the role of this pathway in Arabidopsis, a close relative of *B. napus*. Using an integrated molecular genetic approach, we modulate the availability of the Rubisco substrate ribulose-1,5-bisphosphate (RuBP) via either elimination or overexpression of PRK. We demonstrate the importance of the PRK/Rubisco shunt on seed oil accumulation and we show that Rubisco-mediated CO_2_ recycling is tightly integrated with carbon and energy metabolism in the embryos of green oilseeds.

## Results

### Mutations in PRK are seedling-lethal

We sought to study the influence of the PRK/Rubisco shunt on Arabidopsis seed lipid metabolism using a genetic approach. Rubisco is a multisubunit enzyme consisting of eight catalytic large subunits and eight small subunits. The large subunit is encoded by the plastid genome, and multiple nuclear genes encode the small subunits (Andersson and Backlund, 2008). Given this complexity, direct genetic manipulation of Rubisco is not straightforward. In contrast, PRK, the enzyme that phosphorylates ribulose-5-phosphate (Ru5P) to generate the Rubisco substrate RuBP, is encoded by a single nuclear gene (locus AT1G32060) and thus is much more easily amenable to genetic manipulation. We therefore used *prk* mutants to study the involvement of the PRK/Rubisco shunt in green Arabidopsis seeds.

**Figure 1:**
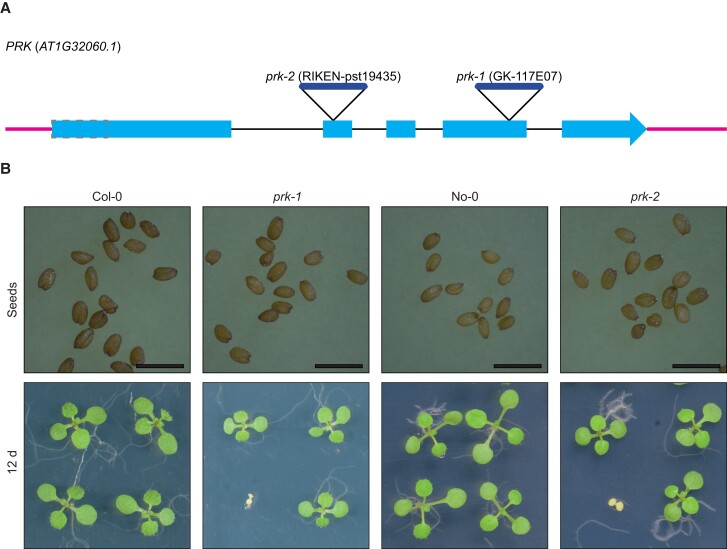
Mutations disrupting PRK are seedling-lethal. A, Structure of the *PRK* locus (AT1G32060.1), depicting untranslated regions (bold magenta lines), introns (thin black lines), and exons (blue boxes). The region encoding the transit peptide of 54 amino acid residues, as predicted by ChloroP (Emanuelsson et al. 1999), is marked with a dashed box. The locations of the T-DNA (*prk-1*) and transposon (*prk-2*) insertions are indicated by triangles. B, Seeds and 12-day-old seedlings derived from selfed heterozygous *prk-1* and *prk-2* mother plants in comparison to the respective wild-type accessions (Col-0 for *prk-1*, No-0 for *prk-2*). The homozygous *prk* seedlings are pale and do not develop beyond the cotyledon stage. Bars = 1 mm.

Previously, PRK was repressed in tobacco (*Nicotiana tabacum* L.) to very low levels, resulting in reductions in CO_2_ assimilation rates, in chlorophyll content, and in relative growth rate (Paul et al., 1995; Banks et al., 1999). These studies suggested that a relatively small fraction of the endogenous PRK is necessary to sustain metabolism under ambient conditions. However, being a core enzyme of the CBBC, the complete loss of PRK should severely compromise plant viability. We obtained two heterozygous Arabidopsis mutant lines, *prk-1* (GK-117E07; a T-DNA insertion line, hereafter referred to as *prk*) and *prk-2* (RIKEN-pst19435; a transposon tagged line). Using PCR-based genotyping and DNA sequencing, we pinpointed their insertions to the fourth (*prk-1*) and second (*prk-2*) exon of *PRK*, respectively (Figure 1A). These heterozygous plants were morphologically indistinguishable from their respective wild types. Both lines produced seeds that were uniform in appearance and germination. However, while three quarters of the seedlings developed in a wild-type-like fashion, one quarter of them became pale immediately after germination and arrested growth at the cotyledon stage of vegetative growth (Figure 1B; Table 1). Using PCR-based genotyping, we confirmed that, as expected, these pale seedlings were homozygous for the T-DNA insertion in the *PRK* locus.

**Table 1:** Segregation analysis of green versus pale seedlings germinating from seeds of a heterozygous *prk-1* mother plant. Seeds were germinated on half-strength MS plates and grown under regular 12-h light/12-h dark cycles for 12 days before classifying them as either green or pale.

Mother plant	Green seedlings	Pale seedlings	Non-germinating	Total
**Col-0**	164 (100%)	0	0	164
**Heterozygous *prk-1***	484 (74.6%)	163 (25.1%)	2 (0.3%)	649

### Generation of lines with visually assessable PRK segregation

Although the striking growth phenotype of the homozygous *prk* offspring became readily apparent after germination, there was no obvious visible effect on the developing seeds of the heterozygous mother plants. In order to study the specific metabolic effects of a loss of PRK, we designed a genetic complementation strategy to be able to identify homozygous *prk* seeds in situ and compare them with their complemented siblings. Therefore, we transformed heterozygous *prk* plants with a construct encoding PRK with a C-terminal YFP tag under the control of the endogenous *PRK* promoter, hereafter referred to as *PRK_pro_:PRK-YFP*. This construct was able to complement the *prk* phenotype: several transgenic lines with a confirmed homozygous *prk* background, but a normal growth phenotype and morphology, could be isolated either directly from the T_1_ or from the T_2_ generation. We applied very stringent selection criteria (see “Materials and Methods” for details) to identify the line best suited for further detailed analysis.

In brief, for each independent transformant line, we screened seedlings on selective media, looking for a segregation ratio of 3:1 (resistant:sensitive), which is indicative of a single-locus insertion of the *PRK_pro_:PRK-YFP* rescue construct. We then used Illumina whole-genome sequencing to simultaneously rule out those with multiple insertions and determine each integration site. Primers specific to these genomic sites and the transgene borders were designed, and the remaining transformants were genotyped to exclude those with tandem inverted insertions. We chose a line with a single-locus transgene insertion in an intergenic region on Chromosome 3 (unlinked to the endogenous *PRK* gene). Finally, we raised antibodies against the recombinant PRK protein and confirmed by immunoblotting that the selected line expressed PRK-YFP to levels similar to the endogenous PRK in the wild-type (Supplemental Figure 1A). Notably, this line, which we refer to as prkCOMP hereafter, has a wild-type-like overall phenotypic appearance (Supplemental Figure 1B).

**Figure 2:**
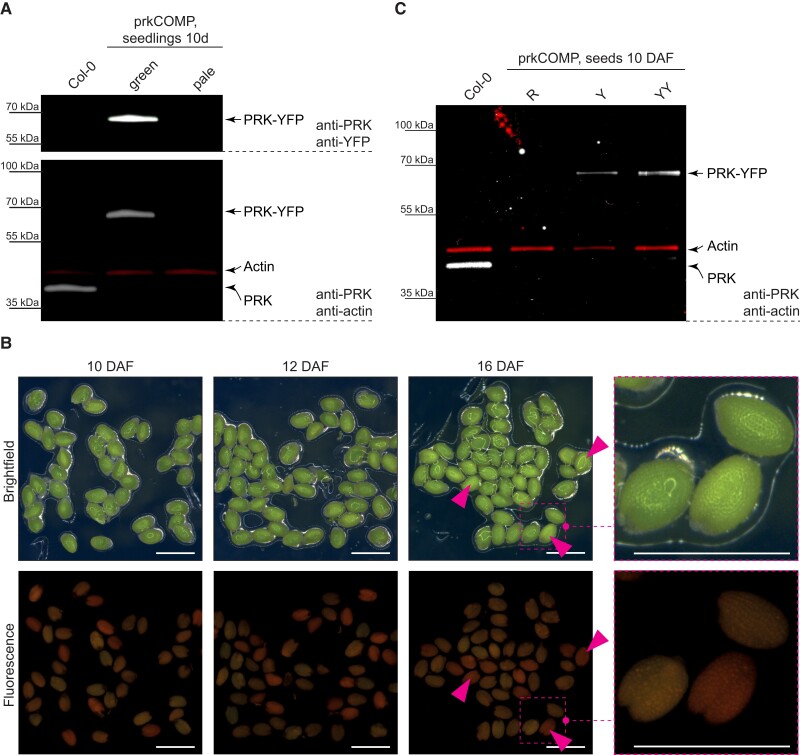
Hemizygous PRK-YFP expression rescues the *prk* growth phenotype and allows selection of homozygous *prk* seeds. A, Immunoblot using extracts of 10-day-old Col-0 WT and prkCOMP seedlings, showing expression of PRK-YFP and lack of endogenous PRK in the segregating population. Seedlings were sorted according to their growth phenotype (green versus pale; compare Figure 1B). Protein was loaded on an equal fresh weight basis (500 ng per lane). The upper blot displays the cumulative signal of two color channels from anti-PRK and anti-YFP antibodies, whereas actin (red signal) was used as a loading control in the lower blot, as marked on the righthand side. B, Segregating populations of green seeds harvested at different stages (DAF: days after flowering) from individual prkCOMP siliques. Seeds classified as “R” according to the fluorescence sorting are marked with magenta arrowheads. Bars = 1 mm. C, Immunoblot of Col-0 WT and sorted green prkCOMP seeds harvested 10 DAF, showing absence of endogenous PRK and presence of PRK-YFP at levels positively correlated with YFP fluorescence in complemented seeds. Protein equivalents to 0.83 seed are loaded per lane; actin (red signal) was used as a loading control, as marked on the righthand side.

We maintained prkCOMP in a hemizygous state for the introduced *PRK_pro_:PRK-YFP* transgene, but homozygous for the *prk* background. Consequently, this line gives rise to seed populations segregating for the complementation construct. We observed that one quarter of the germinated seeds developed into pale, growth-arrested seedlings, as was already observed for the heterozygous *prk* mutant. We confirmed the absence of the endogenous PRK protein and the segregation of the PRK-YFP fusion protein by immunoblotting with antibodies recognizing PRK and the YFP tag (Figure 2A). For this, total protein extracts from both normal green seedlings and pale seedlings derived from prkCOMP were used. The band corresponding to endogenous PRK, visible in the wild-type control, was absent in all prkCOMP-derived seedlings. Both antibodies detected a band at the size expected for the mature PRK-YFP fusion protein in the green prkCOMP-derived seedlings (68 kDa after cleavage of the transit peptide), but not in the pale ones. These results confirmed both the presence of the *prk* mutant background and the successful complementation strategy. Further, the blot using anti-PRK antibodies confirmed again that PRK-YFP was present at comparable levels to the endogenous PRK present in the wild-type seedlings.

We next tested whether we could distinguish between the segregating genotypes at the seed stage based on the fluorescence of the PRK-YFP protein. Indeed, using a simple fluorescence microscope, YFP-fluorescent and non-YFP-fluorescent seeds could readily be distinguished within each silique (Figure 2B). Seeds were sorted into three populations: 23.6% (median ratio of seeds per silique) fluoresced red (R) due to chlorophyll autofluorescence, 56.4% fluoresced yellow (Y), and 20.0% showed high yellow fluorescence (YY). Seeds classified as R presumably represent the noncomplemented *prk* population, while those classified as Y and YY likely correspond to those hemi- or homozygous for *PRK_pro_:PRK-YFP*, respectively. However, the latter sub-classification was more difficult to assign, possibly explaining the slight deviation from the expected number of Y and YY seeds. Nevertheless, immunoblot analyses that showed high and intermediate levels of PRK-YFP protein in extracts prepared from YY and Y seeds, respectively (and none in R seeds), support this assumption (Figure 2C).

### Loss of PRK in developing green seeds depletes Rubisco substrates, leads to reduced lipid accumulation, and affects chlorophyll content

**Figure 3:**
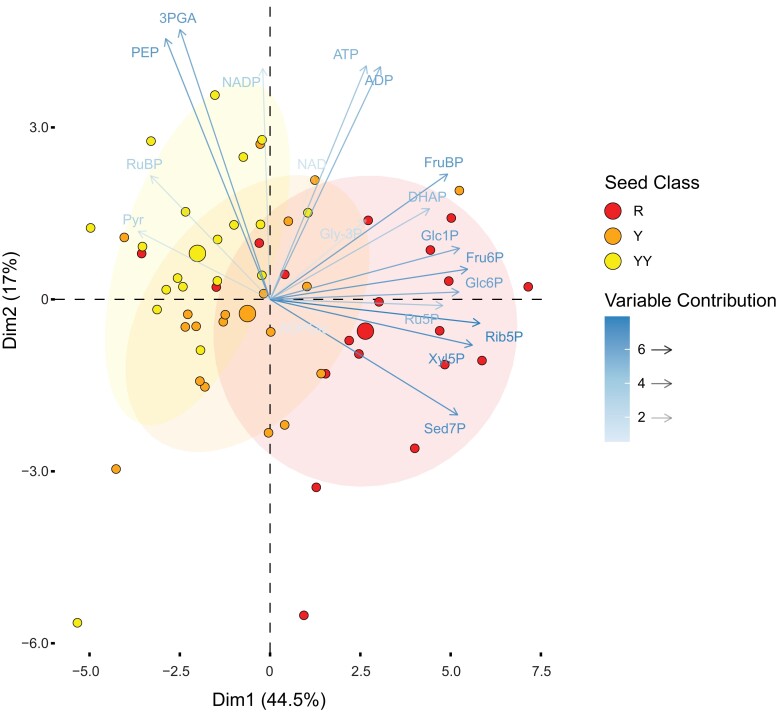
PCA of primary metabolites measured in sorted green prkCOMP seeds. Biplot for principal components 1 and 2. Siliques (*n* = 19) were opened, the seeds therein sorted and pooled according to fluorescence (6–38 seeds per pool sample in the displayed dataset), and metabolites quantified. Small circles represent individual samples and are colored according to their assigned fluorescence class. Large circles represent group mean points and lightly shaded ellipses are concentration ellipses assuming a multivariate normal distribution, drawn to a normal probability of 68%. Metabolite variables are colored and faded according to their contribution. See Supplemental Data Set 1A for raw metabolite data.

We used the segregating offspring from prkCOMP to determine the effects of the absence of PRK on seed metabolites and lipid content. Green developing seeds (10 days after flowering; DAF) were sorted according to their fluorescence as described above. R, Y, and YY seeds from single siliques were separately flash-frozen in liquid N_2_. Samples were extracted and the levels of primary metabolites measured using LC–MS. Clustering of the resulting data via principal component analysis (PCA) revealed that each seed class had a distinct metabolite profile (Figure 3, Supplemental Data Set 1). The R samples clustered apart from the Y and YY samples, which also separate from each other, indicating that the copy number of the *PRK_pro_:PRK-YFP* rescue construct (presumably reflecting the level of PRK activity) influenced the metabolome of the seeds.

**Figure 4:**
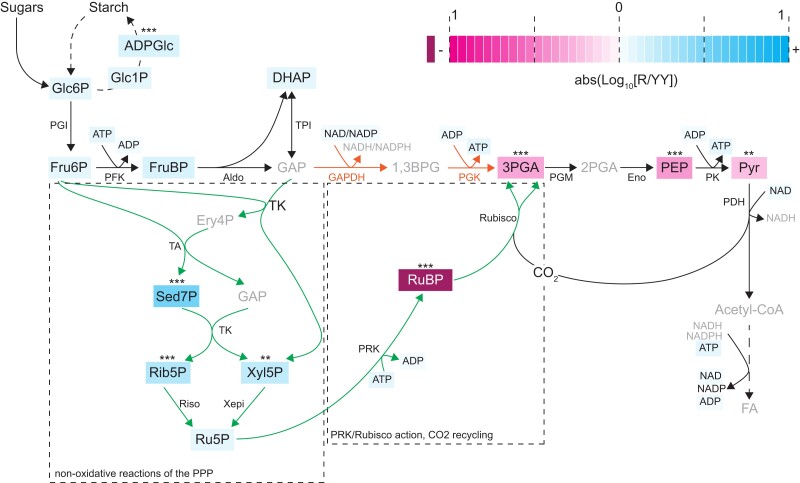
The PRK/Rubisco shunt and changes in the involved metabolites upon loss of *prk* in green seeds. The upper part of the scheme shows the canonical glycolysis pathway. The lower part shows how enzymes of the nonoxidative steps of the pentose phosphate pathway (PPP), together with PRK and Rubisco acting outside the CBBC, bypass parts of glycolysis (indicated in orange) and recycle CO_2_ released by PDH in green Brassicaceae seeds (scheme adapted from Schwender et al., 2004). The relative changes in the mean metabolite levels in R seeds with respect to complemented YY seeds (R/YY; same underlying data as shown in Figure 3) originating from hemizygous prkCOMP mother plants are highlighted with colored boxes (log_10_ scale; see color legend). The value for RuBP was outside the displayed range, with a roughly 19-fold reduction. Statistically significant increases or decreases are indicated by asterisks; **P < 0.01; ***P < 0.001 represent *P* values from two-way ANOVA adjusted for multiple comparisons according to the Šidák method. Metabolites that were not analyzed are displayed in grey. See Supplemental Data Set 1, B and C for statistical analyses and relative metabolite changes, respectively. Metabolites: ADPGlc, ADP-glucose; DHAP, dihydroxyacetone phosphate; Ery4P, erythrose-4-phosphate; FruBP, fructose-1,6-bisphosphate; Fru6P, fructose-6-phosphate; GAP, glyceraldehyde-3-phosphate; Glc1P, glucose-1-phosphate; Glc6P, glucose-6-phosphate; PEP, phosphoenolpyruvate; Pyr, pyruvate; Rib5P, ribose-5-phosphate; RuBP, ribulose bisphosphate; Sed7P, sedoheptulose-7-phosphate; Xyl5P, xylulose-5-phosphate; 1,3BPG, 1,3-bisphosphoglycerate; 2PGA, 2-phosphoglycerate; 3PGA, 3-phosphoglycerate. Enzymes: Aldo, FruBP aldolase; Eno, 2PGA enolase; GAPDH, GAP dehydrogenase; PFK, phosphofructokinase; PGI, phosphoglucose isomerase; PGK, phosphoglycerate kinase; PGM, phosphoglyceromutase; PK, Pyr kinase; Riso, Rib5P isomerase; TK, transketolase; TPI, triose phosphate isomerase; Xepi; Xyl5P epimerase.

We compared the metabolite levels in the R (corresponding to *prk*) and YY (corresponding to fully complemented) seeds. Consistent with a block at the PRK step, the content of several pentose phosphate pathway metabolites upstream of the PRK/Rubisco shunt, notably sedoheptulose-7P (Sed7P), ribose-5P (Rib5P), and xylulose-5P (Xyl5P), were significantly increased in R compared with YY seeds, although surprisingly, the PRK substrate Ru5P was not (Figure 4, Supplemental Data Set 1). Conversely, the PRK product RuBP was barely measurable (below the dynamic range of the calibration curve for all R samples; see “Materials and Methods” section for details), and metabolites of the glycolytic sequence including the Rubisco product 3PGA, PEP, and Pyr, were strongly decreased in R seeds. These data provide direct evidence that PRK and Rubisco are operating in green, developing Arabidopsis seeds.

**Figure 5:**
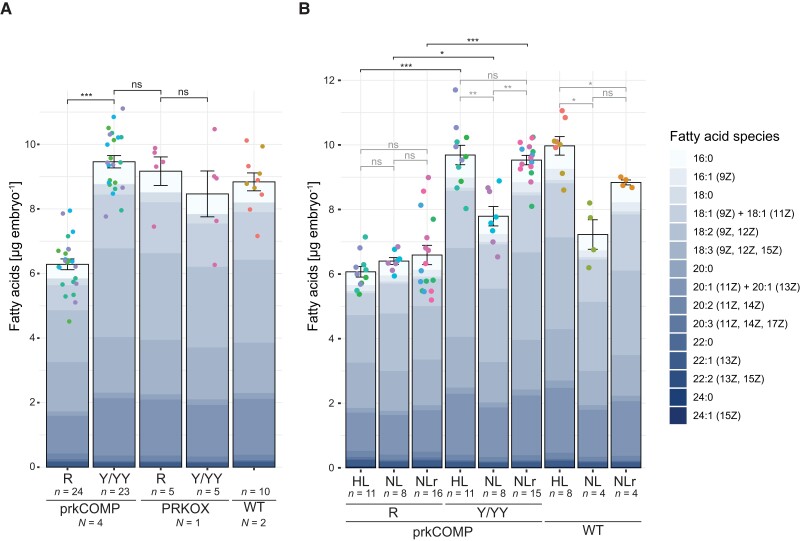
FA quantification in embryos dissected from mature seeds. A, FA contents of sorted embryos excised from prkCOMP, PRKOX, and Col-0 WT seeds. Mature, imbibed seeds were dissected and embryos were sorted according to their fluorescence (no distinction was made between Y and YY samples). FAs were extracted and measured using GC-FID. Values displayed are mean total FA contents on a per-embryo basis (black bar outline) and mean contribution of different FA species to the total content in color. Individual biological replicates (*n*) consisted of pools of several (5–10) embryos. *N* = number of mother plants whose seeds were sampled. Error bars represent the standard error of the mean (SEM). ns, not significant (*P* > 0.05); ***P < 0.001 based on two-tailed *t*-tests for homoscedastic groups. See Supplemental Data Set 2 for FA quantifications and a statistical analysis of total FA contents. B, FA content of sorted prkCOMP and Col-0 WT embryos in relation to light intensity perceived during development. Mother plants were grown at high light intensity (HL: 300 μmol m^−2^ s^−1^). Grey filters reducing light intensity by 50% to normal light (150 μmol m^−2^ s^−1)^ either on only the mother plant's rosettes (NLr samples) or the entire mother plant (including developing siliques; NL samples) were installed near the time of bolting. Mature seeds were dissected, sorted, and extracted as in (A); data display is as in (A). *P < 0.05; **P < 0.01; ***P < 0.001 based on Welch ANOVA with Dunnett's T3 correction for multiple comparisons. See Supplemental Data Set 3, A and B for FA quantifications and a statistical analysis of total FA contents, respectively. In both (A) and (B), individual data points are scattered over the bars, with matching colors representing the same mother plant.

Because Pyr is converted to the FA precursor acetyl-CoA, we next measured if R seeds had reduced levels of FAs in mature embryos. While it was possible to sort seeds according to their fluorescence when they were green, with a translucent seed coat, it was more difficult when the seeds were mature. Here, direct visual phenotyping was prevented by the darkened, pigmented seed coat. Therefore, after a brief (2–4 h at 4°C) period of imbibition, the seed coat was removed prior to fluorescence screening. TAG contents have previously been observed to remain stable for 16 h of imbibition at 4°C (Li et al., 2006). We sorted the embryos according to their fluorescence, which could easily be designated YFP-fluorescent or non-YFP-fluorescent. However, as it was more difficult to distinguish between Y and YY classes, all YFP-fluorescent embryos were pooled (referred to as Y/YY hereafter). Lipids were quantified as fatty acid methyl esters (FAMEs) (Lang et al., 2011) upon methanolysis and were detected using gas chromatography (GC) coupled to a flame ionization detector (GC-FID) (Miquel and Browse, 1992). Remarkably, R embryos had a highly reproducible ∼34% reduction of total lipid content compared with Y/YY embryos (Figure 5A). We also analyzed excised embryos from Col-0 wild-type plants, whose lipid content was in a similar range as that of complemented PRK-YFP embryos, suggesting that our complementation strategy adequately restores a wild-type-like status.

The overall FA profiles were similar to those found in previous reports (Lemieux et al., 1990). However, the absence of PRK disproportionally affected specific FA species. Interestingly, the proportions of 18:1 and 18:2 FAs were significantly reduced in R compared with Y/YY embryos, whereas 18:3 was increased (Supplemental Data Set 2, Supplemental Table 1). The proportions of very long-chain FAs 20:2, 20:3, and 22:1 were increased, although these are minor. In contrast, the proportion of the major FA species 20:1, representing a marker FA of TAG in Arabidopsis (Lemieux et al., 1990), was normal, suggesting that oil storage was not specifically affected.

The proposed contribution of PRK/Rubisco to FA biosynthesis in green seeds is dependent on ATP and reducing equivalents derived from the photosynthetic electron transport chain. Thus, we next tested both whether the intensity of light that siliques were exposed to during seed development influenced the accumulation of lipids, and whether this influence was conditional on the presence of PRK. For this, prkCOMP plants were grown in high light (300 μmol m^−2^ s^−1^; twice the normal the light intensity) and subsequently subjected to three different treatments during seed development: (1) a reduction to normal light (NL: entire plant shielded with a grey filter that reduced light intensity by 50%); (2) normal light for maternal source tissues, high light for siliques (NLr: rosette shielded with a grey filter that reduced light intensity by 50%); and (3) high light (HL: no filter applied). Seeds were harvested, sorted, and total FA contents measured as described above. As expected for a light-supported process, FA accumulation in Y/YY and control Col-0 wild-type embryos positively correlated with the light intensity experienced during seed maturation. Reducing the illumination of the rosette did not influence lipid accumulation in Y/YY, but interestingly, a small reduction was observed for wild-type embryos. This may reflect an influence of maternal *PRK* copy number (two in the wild-type versus the one in prkCOMP) on accumulation of seed storage compounds under higher light. In contrast, the FA content of dissected R embryos did not differ significantly between any of the treatments (Figure 5B, Supplemental Data Set 3). Together, these measurements confirm that the process of lipid biosynthesis in Arabidopsis embryos is supported by light, and that this support is dependent on PRK.

**Figure 6:**
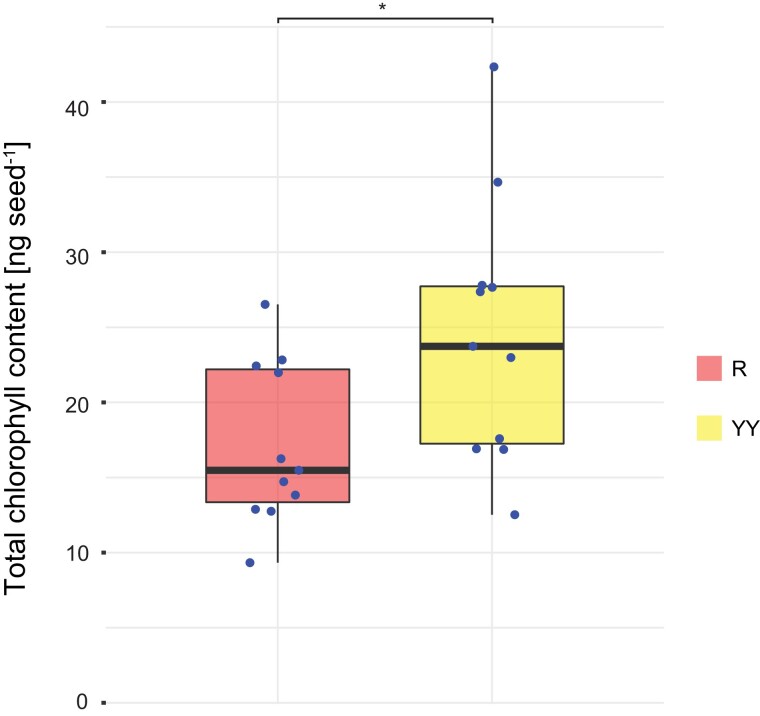
Chlorophyll content of sorted green prkCOMP seeds. Values displayed are the total chlorophyll contents of R and YY seeds. Samples (*n* = 11) consisted of pooled seeds (11–22) from the same silique. The asterisk represents a statistically significant difference (*P < 0.05) between the two groups based on a two-tailed paired *t*-test. See Supplemental Data Set 4 for chlorophyll quantifications and a statistical analysis thereof.

Intriguingly, whenever sorting developing green prkCOMP seeds, we noticed that R seeds, in addition to being devoid of yellow fluorescence, appeared slightly paler than Y or YY seeds under brightfield microscopy conditions (Figure 2B). We therefore measured chlorophyll levels and indeed found that R seeds had a reduced total chlorophyll content compared with their YY siblings (Figure 6, Supplemental Data Set 4). This suggests that the loss of PRK has secondary effects on the photosynthetic capacity.

### Eliminating the flux through PRK/Rubisco perturbs the starch/lipid balance in *prk* embryos

**Figure 7:**
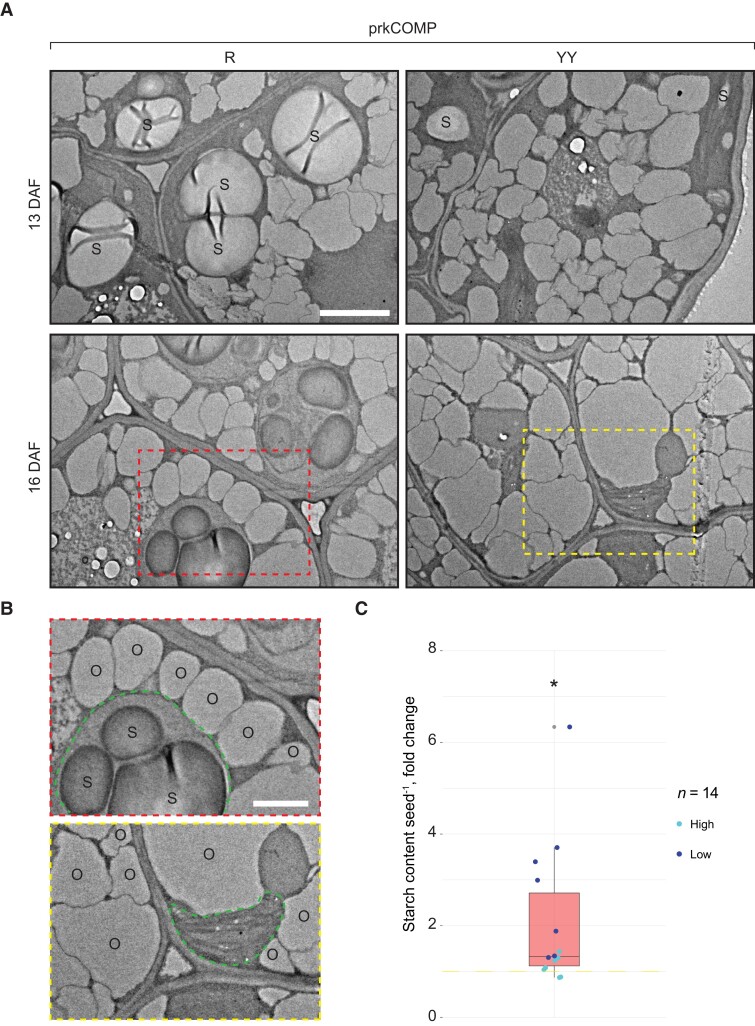
Visualization of lipid bodies and starch in green *prk* and complemented embryos. A, TEMs of thin sections of resin-embedded prkCOMP embryos harvested and dissected 4 h into the day. At 13 DAF, starch granules (S) were present in plastids of both *prk* (R) and the complemented (YY) siblings (upper micrographs; similar observations were made at 7 DAF in another experimental batch). At 16 DAF, starch granules were still present in *prk* embryos, but largely absent in YY embryos (lower micrographs). Bar = 2 μm. B, Enlarged regions of 16 DAF micrographs (from the dotted rectangles in A). Green dashed outlines indicate plastids; S, starch granules; O, oil bodies. Bar = 1 μm. Parts A and B show representative images of >10 images taken for each section. C, Starch measurements of sorted green prkCOMP seeds harvested at 11 DAF. Values displayed are the starch contents of R seeds normalized to the respective YY sample from the same silique. Siliques segregated into two classes with either high- (>15 nmol seed^−1^) or low-starch content, possibly due to small differences in their developmental stages. This classification of samples is indicated by the color of individual data points. Samples (*n*) consisted of pools of seeds (11–22) from the same silique. All siliques originated from the same mother plant. The asterisk represents a statistically significant difference (*P < 0.05) against the null hypothesis *μ*_0_=1 (indicated as yellow dashed line), based on a two-tailed *t*-test. See Supplemental Data Set 5 for starch quantifications and the statistical analysis thereof.

When analyzing our LC/MS measurements on sorted developing prkCOMP seeds, we noticed that the total pool of interconvertible hexose phosphates (Glc6P, Glc1P, and Fru6P) was significantly increased in R seeds relative to YY seeds (See Supplemental Data Set 1). Hexose phosphates not only feed into glycolysis and the PRK/Rubisco shunt, but also supply plastidial starch biosynthesis via the generation of ADPGlc, whose level was also increased (Figure 4). Arabidopsis embryos are known to transiently store starch around 3–14 DAF (Baud et al., 2002), but this starch is almost completely degraded again and replaced by lipids once seeds reach a mature stage (approximately 20 DAF). To assess whether this metabolic branch was affected by the loss of the PRK/Rubisco shunt, we harvested and sorted green prkCOMP seeds at different stages of development, removed their seed coats to facilitate chemical fixation, and embedded them in plastic resin. We then investigated the cellular contents of R and YY embryos using transmission electron microscopy (TEM). As expected from our earlier findings, R embryos had fewer and smaller lipid bodies than did their complemented siblings (Figure 7, A and B). The plastids of both types of embryos still contained starch granules at 13 DAF, but while most of this starch had disappeared in YY embryos at 16 DAF, granules were still present in R embryos, suggesting that the turnover of starch was delayed.

We quantitated the starch content in sorted green prkCOMP seeds in order to substantiate our TEM observations. Starch in the seeds was solubilized, enzymatically digested, and the resulting free glucose was quantified as described previously (Solhaug et al., 2019). Surprisingly, there was appreciable variation in seed starch content, from 3 to 53 (R) and 1 to 50 nmol (YY) glucose equivalents seed^−1^ in the samples, depending on the silique from which they were taken (Supplemental Data Set 5). We assume this was due to slight differences in the developmental stages of the siliques at the time of harvest. To correct for this, we normalized the starch content of each R sample to the value of the respective YY sample from the same silique. The resulting fold changes indicated a significantly increased seed starch content for R seeds (Figure 7C). Together with the increased hexose phosphate and ADPGlc content and the retention of starch granules in TEM observations, this suggests that the biosynthesis of seed starch is upregulated in the absence of PRK. However, it is also possible that starch is not properly mobilized during later development.

### Increased flux through PRK/Rubisco does not translate into increased lipid content

Considering that the absence of PRK resulted in a decreased glycolytic intermediate content, reduced lipid content, and the loss of light-responsive lipid accumulation, we tested whether an increase in PRK abundance would have the opposite effect. We isolated a line carrying the same *PRK_pro_:PRK-YFP* transgene insert as prkCOMP in a hemizygous state in the wild-type genetic background (Supplemental Figure 1B). This line also produces seed populations with observable PRK-YFP segregation, which in this case allowed us to compare metabolite contents of seeds with enhanced PRK expression (because *PRK_pro_:PRK-YFP* is expressed in addition to endogenous PRK) compared with wild-type seeds in the same silique. We refer to this line as PRKOX.

We first assessed metabolite levels in sorted, green PRKOX seeds using LC–MS. Compared with their wild-type siblings, YY PRKOX seeds had highly elevated levels of the PRK reaction product RuBP, suggesting that PRK activity was indeed increased (Supplemental Figure 2, Supplemental Data Set 6). Unexpectedly, the level of the PRK substrate Ru5P was also elevated in the YY seeds. The reason for this is unclear, but some hydrolysis of RuBP to Ru5P may have occurred during extraction and caused this. The accumulation of RuBP to high levels suggests that Rubisco activity may limit the new flux regime. Nevertheless, levels of downstream glycolytic metabolites up to Pyr were increased, suggesting that the flux through the PRK/Rubisco shunt was higher in PRKOX YY seeds than in their wild-type siblings. Interestingly, the level of the starch substrate ADPGlc was again strongly increased. This may be the result of high 3PGA levels stimulating AGPase activity (Iglesias et al., 1993). Interestingly, the increases in ADPGlc, 3PGA, PEP, and Pyr in YY PRKOX seeds was contrasted by decreases in FruBP and Gly-3P (not shown in Supplemental Figure 2; see Supplemental Data Set 6, A–C), suggesting that the substrates of glycolysis may have been depleted by drawing metabolites through the nonoxidative pentose phosphate pathway for the PRK/Rubisco shunt, and their redirection to ADPGlc through AGPase.

We also tested whether the increased flux through the PRK/Rubisco shunt influenced embryo lipid accumulation. Despite the increased abundance of FA precursors in YY PRKOX seeds, the FA content itself was not measurably increased compared with the FA content in their wild-type siblings (Figure 5A), suggesting the presence of additional metabolic bottlenecks downstream in lipid biosynthesis.

## Discussion

### Elimination of PRK causes a seedling-lethal phenotype

PRK has been previously targeted by antisense expression in tobacco and the resulting effects were studied mostly with a focus on overall plant growth and photosynthesis (Paul et al., 1995; Banks et al., 1999). These important studies predate the discovery of the PRK/Rubisco shunt. Also, to our knowledge, the specific effects of PRK deficiency on lipid metabolism in the seeds of these lines has not been assessed. Furthermore, we are not aware of any previous investigations into the phenotype of complete loss-of-function mutants of PRK in vascular plants. Not surprisingly, we found that such mutants fail to complete the transition from heterotrophic to photoautotrophic growth and that their development is thus arrested at the cotyledon stage (Figure 1B), as previously seen for other mutants with an impaired CBBC (Carrera et al., 2021).

To study the specific effects of blocking the PRK/Rubisco shunt on seed lipid biosynthesis, we needed to establish an effective and refined genetic complementation strategy. Our prkCOMP line features several characteristics rendering it a suitable tool for detailed genetic analyses. First, each individual hemizygous prkCOMP mother plant produces both offspring that entirely lack PRK and offspring that express PRK to wild-type-like levels in all of its siliques. This enables the importance of the PRK/Rubisco shunt to be studied independently of maternal- and/or silique-specific effects. This is crucial, because although green seeds are supplied with resources from the mother plant, they utilize external light to support endogenous processes. Thus, their metabolism is intrinsically responsive to both maternal and environmental influences. Indeed, genetic and environmental factors affecting the metabolic status of the mother plant can strongly influence seed storage compound accumulation (Periappuram et al., 2000; Li et al., 2006; Andriotis et al., 2010, 2012). Second, the genotypes of individual offspring developing in prkCOMP siliques can be identified using the YFP tag that is fused to PRK. This allowed us to screen for homozygous rescued seeds and thus minimize differences due to variable PRK expression. Third, similar to the heterozygous *prk* mutants, the prkCOMP line had a growth phenotype similar to wild-type plants, despite carrying only a single allele of the rescue construct (Supplemental Figure 1B). It is unlikely that, under normal growth conditions, photosynthesis is limited by a reduced PRK level resulting from a single gene copy, since early experiments on spinach chloroplasts estimated that PRK reached only a small fraction of its maximal activity under physiological conditions (Gardemann et al., 1983). Later, using antisense tobacco lines, measurable reductions in photosynthesis were detected only when PRK was repressed to very low levels, and decreased enzyme abundance correlated with a compensatory increase in its activation state (Paul et al., 1995; Banks et al., 1999). Thus, we can largely exclude the possibility that the prkCOMP line is impaired in providing substrates to its seeds under normal growth conditions, though it remains possible that there is a small effect when grown in high light.

### The reduction in lipid content in prk embryos exceeds that predicted by the *PRK*/RUBISCO shunt

Despite the severe effect on seedling growth, the loss of PRK did not visibly affect either seed development or final seed size (Figure 2B). Nevertheless, its loss essentially eliminated the Rubisco substrate RuBP (Figure 4) and blocked the proposed glycolytic bypass acting during lipid synthesis in green seeds. As expected, we observed accumulation of the upstream pentose phosphate pathway metabolites, and downstream FA precursors were depleted. Furthermore, there was a marked decrease in lipid content in mature embryos (Figures 4 and 5). On one hand, this confirms that PRK and Rubisco add to the efficiency of storage lipid metabolism in green Arabidopsis seeds. On the other hand, the extent to which this occurs was surprising: Mature *prk* embryos contained 34% less total FA than their PRK-expressing siblings. Interestingly, this is roughly two times higher than earlier estimates of the contribution of the PRK/Rubisco shunt in Arabidopsis (Lonien and Schwender, 2009), and still 1.7 times the maximum that the shunt could theoretically provide, assuming that all imported carbon directed into acetyl-CoA would flow through Rubisco. Because oil represents a major component of mature Arabidopsis seeds (36% by weight; Li et al., 2006), we would expect the observed reduction in FA content to translate into a decreased overall embryo weight, and that the magnitude of decrease would depend on whether any of the carbon originally destined for FA biosynthesis had been redirected into other compounds. However, we did not assess total mature embryo biomass in our experiments, because the prkCOMP seeds can only be sorted at this stage after removal of the seed coat, necessitating seed imbibition.

Our data provide further insight that allows us to propose explanations for this unexpectedly strong effect. The activity of the PRK/Rubisco shunt depends on a functional photosynthetic transport chain, and thus the availability of light. Indeed, lipid accumulation in green Brassicaceae seeds has been previously linked to the intensity of illumination experienced by the seed during development (Ruuska et al., 2004). Our experiments applying different intensities of light to maturing Arabidopsis seeds confirm this dependence and show that it is lost in the absence of PRK (Figure 5B). Although the PRK/Rubisco shunt uses only a fraction of the energy needed to operate the CBBC, it is still likely to be a major sink for the products of the photosynthetic electron transport chain in green seeds, together with downstream steps in FA biosynthesis. The loss of PRK could therefore result in an excess of energy and reductant generated by the photosynthetic light reactions; no ATP would be needed for the PRK step, and the depletion of precursors would reduce the rate of FA and thus the rate of lipid biosynthesis. Consequently, excessive reduction of the photosynthetic electron transport chain may occur, with enhanced generation of reactive oxygen species (ROS) and resultant photo-oxidative damage and bleaching (Niyogi, 1999). This is clearly evident in the cotyledons of germinated *prk* seedlings as a result of their inability to run the CBBC. It may also occur in developing *prk* seeds—those originating from our prkCOMP line appeared paler and had less chlorophyll compared with their complemented siblings (Figures 2B and 6). If *prk* seeds indeed suffer from an imbalance between photosynthetic light harvesting and utilization of this energy, there may be further unanticipated pleiotropic effects contributing to the reduced accumulation of storage compounds.

Our metabolite measurements do not provide sufficient information to assess metabolic fluxes. Nevertheless, the unchanged levels of glycolytic substrates between complemented and noncomplemented *prk* seeds (Figure 4) allow us to speculate that one or more of the early steps of glycolysis may have insufficient capacity to channel all carbohydrates obtained from the mother plant to FA biosynthesis. Furthermore, one could imagine that curbing the activity of glycolysis was a necessary evolutionary step to facilitate the alternative flux through the pentose phosphate pathway and PRK/Rubisco shunt, given that the two pathways draw from the same pool of resources. The fact that we also detected an increase in the total pool of hexose phosphates in *prk* seeds supports the idea that maternally derived carbohydrates are not efficiently used. This side effect could further explain our observation that green *prk* seeds have elevated ADPGlc, and at least transiently produce/retain higher levels of starch compared with their PRK-containing siblings; transiently elevated starch levels were observed in green seeds of the Arabidopsis *wri1* mutant, which feature increased soluble sugar contents alongside much reduced FA biosynthesis (Focks and Benning, 1998).

Consistently, a recent study of in vitro cultured *B. napus* embryos demonstrated the existence of a trade-off between the partitioning of carbon into starch versus lipids (Schwender et al., 2015). Thus, the blockage in the PRK/Rubisco shunt, together with the increased pool of hexose phosphates, could redirect resources into starch production, further helping to explain the large decrease in partitioning to lipid biosynthesis. However, since starch is synthesized and then degraded within a relatively short time frame during embryo development (Baud et al., 2002), further time-course measurements are needed to provide quantitative information about the changes in carbon allocation to the lipid and starch pools in *prk* seeds, and to understand how this translates into the final starch and lipid composition in the mature seed.

We also cannot exclude the possibility of an active CBBC cycle operating to some extent in developing Arabidopsis embryos. Evidence of CBBC activity in isolated *B. napus* embryos was observed under in vitro conditions at a light intensity of 150 μmol m^−2^ s^−1^ (Goffman et al., 2005). Although this is the same light intensity that we used for our experiments, we would expect the silique wall and seed coat to significantly reduce the light reaching the embryos—only 20%–30% of the light is transmitted in the case of *B. napus* (Eastmond et al., 1996; King et al., 1998). It is presently unclear whether these intensities would be sufficient to sustain CBBC activity in vivo. However, any CBBC activity would be abolished in *prk* embryos in addition to the PRK/Rubisco shunt, and this could help explain the higher-than-expected influence on seed oil accumulation.

Finally, it is also worth noting that seed production is energetically costly to the mother plant, and its limited resources are divided among the whole population of developing seeds. We cannot exclude the possibility that lower utilization of imported carbohydrate in green *prk* seeds negatively affects resource import (e.g. if sucrose accumulates and inhibits further uptake). Indeed, significantly reduced sucrose uptake has been observed for Arabidopsis *wri1* mutant embryos cultivated in vitro (Lonien and Schwender, 2009). If so, the maternal allocation to seeds of different genotypes may vary, with resources diverted to those seeds capable of utilizing the substrates more efficiently, i.e. those with a stronger sink strength. Such a scenario may combine two effects: first, reduced lipid accumulation in *prk* seeds; and second, increased lipid accumulation in complemented siblings. Such a shift in resource allocation, though likely to be small, may contribute to the larger-than-expected difference in oil content measured between *prk* and wild-type-like embryos.

### Enhancing the flux through PRK is insufficient to increase lipid accumulation

As a complement to prkCOMP, the PRKOX line allowed us to assess the effects of increased PRK activity in the developing seed. The accumulation of RuBP in YY seeds of this line suggests that Rubisco activity could not keep up with the increased PRK activity and may represent one of the next limiting factors in the pathway. This is somewhat surprising, because transcriptome data from developing Arabidopsis seeds indicated that expression of both PRK and Rubisco are induced at the same time as are many enzymes involved in FA biosynthesis (Ruuska et al., 2002). Furthermore, Rubisco was not only abundant but also highly carbamylated (and thus active) in developing *B. napus* embryos, presumably due to the prevailing high concentration of CO_2_ (Ruuska et al., 2004; Hajduch et al., 2006). However, it is still possible that the increase in PRK activity in our PRKOX line is eventually saturating for Rubisco; indeed, we did observe some increases in 3PGA, PEP, and Pyr levels (Supplemental Figure 2), indicating that the PRK/Rubisco shunt may have been more active. The fact that this did not translate to a measurable increase in lipid content may indicate that other steps, possibly located in the downstream FA biosynthesis or subsequent TAG assembly pathways, also limit lipid accumulation (Bates et al., 2013).

Much evidence points toward lipid accumulation being a complex and regulated metabolic process that is reliant on a multitude of enzymatic and environmental factors. Several approaches individually targeting suspected rate-limiting steps of FA biosynthesis had only modest effects, no effects, or even a negative impact on seed oil accumulation in *B. napus* (Verwoert et al., 1995; Roesler et al., 1997; Dehesh et al., 2001). Although in a recent study, seed oil content was significantly increased by overexpression of pea (*Pisum sativum*) α-carboxyltransferase, a subunit of ACCase, in Arabidopsis and camelina (*Camelina sativa*) (Wang et al., 2022), top-down metabolic control analysis suggested that not only plastidial FA biosynthesis, but also the ER-located Kennedy pathway exert control over lipid biosynthesis in oil palm (*Elaeis guineensis* Jacq.) and olive (*Olea europaea* L.) (Ramli et al., 2002). Indeed, overexpression of an Arabidopsis Gly-3P acyltransferase (GPAT9), a yeast (*Saccharomyces cerevisiae*) *lyso*-phosphatidic acid acyltransferase (LPAT), and diacylglycerol acyltransferase (DGAT), catalyzing the first, second, and final steps of TAG formation in the ER, respectively, increased seed oil content in *B. napus* and Arabidopsis (Zou et al., 1997; Jako et al., 2001; Taylor et al., 2002; Singer et al., 2016). Increasing the levels of Gly-3P (which forms the backbone of TAGs) by overexpression of a yeast Gly-3P dehydrogenase was also reported to increase oil content (Vigeolas et al., 2007). This is interesting, as we observed that Gly-3P levels were significantly reduced in YY PRKOX seeds (Supplemental Data Set 6, A–C), and this could limit lipid accumulation despite the accumulation of precursors for FA biosynthesis. Recent reports further indicate that enhanced FA trafficking from the plastids into the ER promotes seed lipid accumulation in Arabidopsis (Kim et al., 2013; Tian et al., 2018; Li et al., 2020).

Several observations suggest that more integrated modifications to seed carbon metabolism can have large influences on seed FA metabolism. Studies of the transcription factor WRI1 are illustrative of this phenomenon. Seeds of *wri1* show a drastic reduction in seed oil content alongside accumulation of soluble sugars, likely as a consequence of a largely affected transcriptional program that results in altered activities of the enzymes involved in glycolysis and FA biosynthesis (Focks and Benning, 1998; Ruuska et al., 2002; Baud and Graham, 2006; Baud et al., 2007). Constitutive and ectopic overexpression of WRI1 in Arabidopsis increased seed oil content, but also caused the accumulation of TAG in seedlings grown in the presence of exogenous sugar sources (Cernac and Benning, 2004). Interestingly, the broad effects of WRI1 overexpression appear to be even more exploitable by simultaneous upregulation of TAG assembly (via DGAT expression) and disruption of TAG turnover (via lipase suppression), with the combined effect being stronger than either approach alone (Van Erp et al., 2014). Together, this indicates that the coordinated modulation of several factors and steps of lipid biosynthesis may be the most promising approach to increase seed oil content, which is consistent with our observations regarding the PRKOX line.

In conclusion, our observations provide genetic confirmation for the operation of the PRK/Rubisco shunt, and suggest that this pathway is tightly integrated with light harvesting and starch biosynthesis in the Arabidopsis embryo. We also show a potential for increasing the flux through the PRK/Rubisco shunt to provide more FA precursors, which, combined with other targeted interventions, could help increase lipid production in commercially important green oilseed species.

## Materials and methods

### Plant material and growth conditions

Two independent *PRK* (AT1G32060) insertional mutants of *A. thaliana* (Arabidopsis) were obtained from the European Arabidopsis Stock Centre. The *prk-1* T-DNA insertion mutant is from the GABI-KAT collection (GK-117E07) and was generated in the Columbia (Col-0) ecotype background. The *prk-2* transposon tagged line is from the RIKEN collection (RIKEN_pst19435) and is in the Nossen (No-0) ecotype background. The T-DNA and transposon insertions in exons four and two, respectively, were confirmed by PCR-based genotyping followed by Sanger sequencing of the resulting DNA bands.

For the assessment of seedling development, seeds were surface sterilized by a series of washes with first 4% [v/v] bleach and 0.6% [v/v] Tween20, then 70% [v/v] ethanol, and finally sterile water. Seeds were subsequently resuspended in 0.1% [w/v] agar before being plated on a solidified half-strength Murashige and Skoog (MS) medium. Seeds were stratified for 2 days at 4°C and subsequently grown for 12 days in regular 12-h light and 12-h dark cycles before phenotypic scoring.

Plants were grown on standard soil (Substrate 2 from Klasmann-Deilmann GmbH, Geeste, Germany). No additional fertilizer was applied during growth. The plants were grown in a Kälte 3000 chamber illuminated with fluorescent bulbs (Master TL5 HO 39W/865, Philips, Amsterdam, Netherlands) as the light source, under long-day conditions (16 h light per day), 150 μmol m^−2^ s^−1^ of light intensity (normal light) unless otherwise specified, at 20°C and 60% relative humidity. Seeds were collected using clear Aracon tubes unless otherwise specified (Arasystem, Ghent, Belgium), thus avoiding silique shading throughout development.

### Construction of transgenic lines and screening

For cloning of the *PRK_pro_:PRK-YFP* complementation construct, the promoter of *PRK* (comprising 1.9-kb sequence upstream of the translation start at the AT1G32060 locus) was cloned into a Gateway® pDONRTM P4P1r entry vector. The *PRK* coding sequence (excluding the stop codon) was cloned into a Gateway® pDONRTM 221 entry vector. The coding sequence for eYFP, cloned into a Gateway® pDONRTM P2rP3 entry vector, was as in Beeler et al. (2014). Entry vectors were then recombined into the Gateway® destination vector pB7m34GW,0, which contains a glufosinate resistance gene as a selectable marker. The final *PRK_pro_:PRK-YFP* expression vector was transformed into the *Agrobacterium tumefaciens* strain GV3101. Heterozygous *prk-1* plants were transformed using the floral dip method (Clough and Bent, 1998; Zhang et al., 2006), and transformants were selected using the glufosinate resistance marker of *PRK_pro_:PRK-YFP*. Transgenic lines were pre-selected for a glufosinate resistance segregation pattern of 3:1 (resistant:susceptible), consistent with a single-locus T-DNA insertion in the T2 generation. Genomic DNA was extracted from T1 plant material and used for Illumina whole-genome sequencing. A genome coverage of over 97% for all analyzed lines was obtained, and overlapping reads containing both T-DNA borders and sequences matching the Col-0 reference genome were used to identify the loci of insertion. This was successful for four lines—three were found to have the T-DNA inserted in a single genetic locus, and one had T-DNA inserted at two loci with a strong genetic linkage. Molecular markers for confirming the insertion sites and allowing classification at greater detail were designed for the first three lines. This analysis allowed us to exclude another line due to the presence of a tandem insertion. Finally, for all further experiments, we selected the one of the remaining two lines whose insertion was at the greater distance from neighboring genes. The transgene insertion in this line is localized in an intergenic region 1,258 base pairs (bp) upstream of the translation start of *AT3G24800*, which encodes pyruvate phosphate dikinase (PPDK).

### Seed dissection and screening

To discriminate *prk* embryos from embryos complemented with *PRK_pro_:PRK-YFP*, dry mature seeds were imbibed for 2–4 h at 4°C in water, allowing us to remove the seed coats using tweezers. The fluorescence status of each embryo was identified using a Leica zoom microscope equipped with a GFP-LP filter (Leica #10447407, Filter set ET GFP-LP—M205FA/M165FC, excitation 460–500 nm, emission 510 nm long-pass). Dissected embryos were kept on filter paper placed on an agar plate containing half-strength MS medium until further processing.

### Purification of recombinant PRK protein for antibody generation

The *PRK* coding sequence, excluding the first 162 bp predicted to encode a transit peptide (Emanuelsson et al., 1999) and the stop codon, was PCR-amplified using primers containing *Eco*RI and *Xho*I restriction sites. The resulting DNA fragment was then cloned into the pET-21a vector (Novagen) in frame with sequence encoding a C-terminal 6XHis-tag, using its flanking restriction sites. Because early reports have indicated difficulties when expressing wild-type PRK in *Escherichia coli* (Hudson et al., 1992), we performed site-directed mutagenesis using the QuikChangeTM Site-Directed Mutagenesis Kit (Agilent, Santa Clara, USA) to replace the two redox-sensitive Cys residues (Porter and Hartman, 1990; Milanez et al., 1991) with Ser residues, and then transformed *E. coli* BL21 cells for heterologous expression.

Transformed cells were grown at 37°C and 260 rpm (Infors HT Multitron incubator) in LB medium containing ampicillin (50 μg mL^−1^) and chloramphenicol (12.5 μg mL^−1^) to an optical density (OD_600_) of 0.5–0.8. Protein expression was induced by adding isopropyl *β*-d-1-thiogalactopyranoside (IPTG) to a final concentration of 1 mM, after which cells were incubated at 20°C for another 16 h. Cells were then pelleted by centrifugation for 10 min at 4°C and 3,107*g* and then resuspended in lysis buffer (50 mM Tris–HCl, pH 8, 300 mM NaCl, 40 mM imidazole, 2 mM dithiothreitol [DTT], 1 mg mL^−1^ lysozyme and 1X cOmpleteTM Protease Inhibitor Cocktail [Roche, Switzerland]). Cells were passed through a microfluidizer three times and the resulting lysate was cleared by centrifugation for 10 min at 4°C and 20,400*g*. The supernatant was incubated for 1 h with Protino® Ni-NTA Agarose (Macherey-Nagel, Düren, Germany). After a 1-min centrifugation at 4°C and 200*g*, the agarose resin was resuspended in lysis buffer and washed five times with 50 mM Tris–HCl, pH 8, 300 mM NaCl, 40 mM imidazole, 2 mM DTT, 0.5% [w/v] Triton X-100 and then five times with 50 mM Tris–HCl, pH 8, 300 mM NaCl, 40 mM imidazole, 2 mM DTT. Bound protein was eluted with elution buffers containing 50 mM Tris–HCl, pH 8, 50 mM NaCl and a step-wise increasing concentration of imidazole (100, 250, and 500 mM). Fractions for which protein could be detected by Coomassie Brilliant Blue polyacrylamide gel electrophoresis (PAGE) were pooled and concentrated using Amicon® Ultra Cell® 3k centrifugal filter units. Proteins were exchanged into a buffer containing 50 mM Tris–HCl, pH 8, 10% [v/v] glycerol and 2 mM DTT using NAPTM-5 (GE Healthcare Life Sciences, Pittsburgh, USA) columns and were then stored at −80°C.

Rabbits were immunized using the recombinant protein as antigen by Eurogentec (Seraing, Belgium). Antibodies were purified from serum using a two-step procedure first enriching total IgG using Protein A-Agarose (Roche, Switzerland) and then selecting anti-PRK antibodies by affinity purification using recombinant PRK protein coupled to an NHS-activated High Performance column (GE Healthcare Life Sciences, Pittsburgh, USA). Antibodies were concentrated using Amicon® Ultra Cell® 3k spin-filter units (Merck, Germany) and subsequently stored at −80°C in 2.7 mM potassium chloride, 10 mM sodium hydrogen phosphate, 1.8 mM potassium dihydrogen phosphate, pH 7.4. Purified antibodies were used at a dilution of 1:600 for immunoblots (see below).

### Protein extraction and immunoblotting

Plant material was homogenized in 500 mM Tris–HCl, pH 6.8, 30% [v/v] glycerol, 20% [w/v] SDS, 1 M DTT, 0.05% [w/v] bromophenol blue in a buffer-to-tissue ratio of 10 μL per mg fresh weight. After centrifugation (10,000*g*, 5 min, 4°C), the supernatant was kept at −20°C. Proteins were separated by SDS-PAGE and transferred to Immobilon-FL polyvinylidene fluoride (PVDF) membranes (pore size 0.45 μm, Merck Millipore Ltd.). Membranes were dried briefly, reactivated in methanol, and blocked in TBST (20 mM Tris–HCl, pH 7.4, 150 mM glycine, 0.1% [v/v] TWEEN® 20) supplemented with 3% [w/v] skimmed milk powder for 1 h at 4°C. Membranes were then incubated in TBST supplemented with 3% [w/v] skimmed milk powder and appropriate primary antibodies (purified custom anti-PRK: 1:600; commercial GFP antibody: Clontech JL-8: 1:5,000; commercial anti-plant-actin: Sigma–Aldrich, clone 10-B3: 1:10,000) for 16 h at 4°C, washed five times with TBST at room temperature, and incubated for 1 h at RT in TBST supplemented with 3% [w/v] skimmed milk powder, 0.1% [w/v] SDS, and secondary antibodies (IRDye 680RD Donkey anti-Mouse IgG/IRDye 800CW Donkey anti-Rabbit IgG at a dilution of 1:20,000). Membranes were then washed five times with TBST and imaged with a Li-Cor Odyssey System, allowing the fluorochromes coupled to the secondary antibodies to be detected simultaneously.

### Metabolite extraction and LC–MS measurements

Green seeds from single siliques were screened, collected in Eppendorf tubes, and deep-frozen in liquid N2. Samples were homogenized in 250 μL of chloroform:methanol 3:7 [v:v] mixture using a plastic pestle and incubated for 2 h at −20°C, with brief vortexing every 30 min. Three extractions with each 400 μL of H_2_O, each followed by centrifugation (16,000*g*, 5 min, 4°C), were done on each sample, the aqueous supernatants collected and dried using an Eppendorf Concentrator plus (30°C), and stored at −80°C until the measurements (Arrivault et al., 2009). Samples were then dissolved in 100 μL H_2_O, filtered through a 0.2-μm Minisart® RC4 Regenerated Cellulose Syringe Filter (Sartorius AG, Germany), and analyzed using a UHPLC–MS/MS configuration consisting of a 1290 Infinity UHPLC (Agilent, Santa Clara, USA) equipped with an Acquity T3 end-capped reverse phase column (Waters, Milford, USA) that was coupled to a QTRAP 5500 triple quadrupole MS (AB Sciex, Framingham, USA) (Buescher et al., 2010).

Data were analyzed using Multiquant 3.03, ABSciex and relevant metabolites selected for further analysis. Among these, metabolite contents below the dynamic range of the calibration curve were utilized as such, whereas those for which no peak could be integrated were set to the minimum value of the entire dataset times 10^−3^ to allow log_10_-transformation (see Supplemental Data Sets 1 and 6 for details; also note that the metabolite analyses for prkCOMP and PRKOX originate from the same dataset and thus share the same minimum value). Log_10_-transformed data were used for PCA using the R packages FactoMineR (Lê et al., 2008) and factoextra (Kassambara and Mundt, 2017), and for statistical analyses using GraphPad Prism, version 9.3.1, for macOS.

### Lipid quantification

FAMEs were prepared to perform an absolute quantification of the lipid content according to Miquel and Browse (1992). From mature dry seeds, dissected material was transferred into a mixture of methanol:toluene 2:1 [v:v], 2.5% [v:v] H_2_SO_4_, 2% [v:v] dimethoxypropane containing 20 μg of tripentadecanoate (Merck KGaA, Darmstadt, Germany) as internal standard for later quantification. After creating an argon atmosphere, tubes were heated to 80°C for 60 min. Cooled samples were neutralized with 100 mM Tris–HCl, pH 8, 0.9% [w/v] NaCl. After two extractions with hexane, the pooled supernatants were dried under a nitrogen flow and dissolved in 100 μL of acetonitrile. FAME extracts were subsequently separated by GC on a DB-23 column (30 m × 0.25 mm; 0.25 µm coating thickness; J&W Scientific, Agilent, Waldbronn, Germany) followed by detection using a flame ionization detector (FID) according to Lang et al. (2011). The GC program was as follows: 1 min at 150°C, increase to 200°C at a rate of 8°C min^−1^, increase to 250°C at a rate of 25°C min^−1^. Total FA content is defined as the sum of 17 FAME species: 16:0, 16:1 (9*Z*), 18:0, 18:1 (9*Z*), 18:1 (11*Z*), 18:2 (9*Z*, 12*Z*), 18:3 (9*Z*, 12*Z*, 15*Z*), 20:0, 20:1 (11*Z*), 20:1 (13*Z*), 20:2 (11*Z*, 14*Z*), 20:3 (11*Z*, 14*Z*, 17*Z*), 22:0, 22:1 (13*Z*), 22:2 (13*Z*, 15*Z*), 24:0 and 24:1 (15*Z*). Identification of individual FAME species was done using a custom standard FAME mixture (FAME Mix, C4-C24, Merck KGaA, Darmstadt, Germany). FAME quantifications were calculated from extrapolation based on the internal standard.

### Chlorophyll measurements

Chlorophylls from pooled seeds were extracted in 80% [v/v] acetone at 1 µL seed^−1^ for 24 h in the dark at 4°C. Absorbances at 664 and 647 nm were then measured in the supernatant using a nano-drop spectrophotometer (NanoDrop ND-1000) set to UV/Vis mode, using 80% [v/v] acetone as a blank. Total chlorophyll was calculated from A_664_ and A_647_ as described previously (Inskeep and Bloom, 1985). The remaining acetone was then removed and the seed samples were used for starch measurements (see below; samples were stored at −20°C until further processing).

### Starch measurements

Seeds were ground in 40 µL of H_2_O, then two 10 µL aliquots per sample were separately incubated at 100°C for 10 min. Samples were allowed to cool to room temperature (∼22°C) for 10 min before addition of 10 µL of AA/AMG solution to one (digest) and 10 µL of control solution to the other (control) aliquot of each original sample. AA/AMG solution consisted of 2.6 µL (∼26 units) of α-amylase (Roche) and 23.4 µL (∼3.3 units) of amyloglucosidase (Roche) in a final volume of 520 µL with 50 mM sodium acetate, pH 4.8, and the control solution contained 50 mM sodium acetate, pH 4.8 with water added at the same ratio as enzyme for AA/AMG. Digested samples and undigested controls were then incubated for 4 h at 37°C before quantification of free glucose (Glc) using glucose oxidase (GlcOx), horse radish peroxidase (HRP), and Ampliflu-red (Amp-Red) as described previously (Solhaug et al., 2019). The Amp-Red glucose assay solution was prepared by combining 1 µL of Amp-Red (Sigma; 10 mM dissolved in DMSO), 1 µL of GlcOx (Fluka; 0.1 units), 1 µL of HRP (Fluka; 0.1 units), 5.6 µL of sodium phosphate buffer (150 mM, pH 7.4), and made up to 25 µL with DI H_2_O. Samples were mixed with 150 mM sodium phosphate pH 7.4, H_2_O, and Amp-Red glucose assay solution at a 1:1:1:1 ratio. After combining all components, reactions were incubated at room temperature (∼22°C) in the dark for 25 min before measuring absorbances at 570 nm (A_570_). Due to the segregation into high- and low-starch content classes, absorbances had to be measured using different devices. Samples with low glucose content were measured using the UV/Vis function of a nano-drop spectrophotometer, whereas samples with high glucose content were measured using a microplate reader (Tecan infinite M1000). The quantity of Glc produced from starch was determined from the ΔA_570_ of the respective measurements of digested and undigested control samples, using a standard curve of D-glucose (0–500 µM) as a reference.

### Sample preparation for TEM and imaging

Green embryos were sorted, chemically fixed in 100 mM Na-cacodylate, pH 7.4, 2.5% [v/v] glutaraldehyde, and 2% [w/v] formaldehyde. Samples were washed three times with 100 mM Na-cacodylate, pH 7.4, and then stained with 1% [w/v] osmium (VIII) tetroxide in 100 mM Na-cacodylate, pH 7.4. Samples were washed with H_2_O and dehydrated in an aqueous dilution series of ethanol (50%, 60%, 70%, 80%, 98%, 3 × 100%). Samples were then infiltrated with Spurr (Polysciences) resin (2 × 25%, 2 × 50%, 2 × 75%, 3 × 100%; Spurr dilutions in dehydrated ethanol). All steps were done using a Pelco Biowave® Pro+. Resin blocks were polymerized at 60°C for 2 days, and 70 nm thin sections cut using an ultramicrotome (Leica Ultracut E). Sections were incubated with 2% [w/v] uranyl acetate and Reynold's solution (1.33 g Pb(NO_3_)_2_, 1.76 g Na_3_ (C_6_H_5_O_7_) 2H_2_O, 30 mL distilled water; Reynolds, 1963) for 10 min each, with four washes of water in between and afterwards. TEM micrographs were acquired using a JEOL JEM-1400 Flash Electron Microscope.

### Accession numbers

Arabidopsis *PRK,* AT1G32060. Germplasm used: *prk-1* T-DNA insertion mutant, GK-117E07, Columbia (Col-0) background; *prk-2* transposon mutant, RIKEN_pst19435, Nossen (No-0) background.

## Supplemental Data

The following materials are available in the online version of this article.

**Supplemental Figure 1.** Near-endogenous expression levels of PRK-YFP in 1-month old prkCOMP rosette leaves, and prkCOMP/PRKOX growth (supports Figure 2A and C).

**Supplemental Figure 2.** The PRK/Rubisco shunt and changes in the involved metabolites upon overexpression of PRK in green embryos (supports Figures 4 and 5A).

**Supplemental Table 1.** Analysis of individual FA species in sorted prkCOMP embryos.

**Supplemental Table 2.** Oligonucleotide primers used in this study.

**Supplemental Data Set 1.** LC–MS based quantification of selected metabolites in green prkCOMP seeds.

**Supplemental Data Set 2.** FA quantification in sorted, mature WT, prkCOMP, and PRKOX embryos.

**Supplemental Data Set 3.** FA quantification in sorted, mature prkCOMP embryos grown under different light conditions.

**Supplemental Data Set 4.** Chlorophyll measurements of sorted, green prkCOMP seeds.

**Supplemental Data Set 5.** Starch content measurements of sorted, green prkCOMP seeds.

**Supplemental Data Set 6.** LC–MS based quantification of selected metabolites in green PRKOX seeds.

## Supplementary Material

koac338_Supplementary_Data
