# Supplementary material for: The PRK/Rubisco shunt strongly influences Arabidopsis seed metabolism and oil accumulation, affecting more than carbon recycling
Source: Plant Cell. 2022 Dec 1;35(2):808–26. doi: 10.1093/plcell/koac338 (PMC9940875; doi:10.1093/plcell/koac338)
Supplement: koac338_Supplementary_Data [file koac338_supplementary_data.zip › SUPPLEMENTAL DATA.pdf]

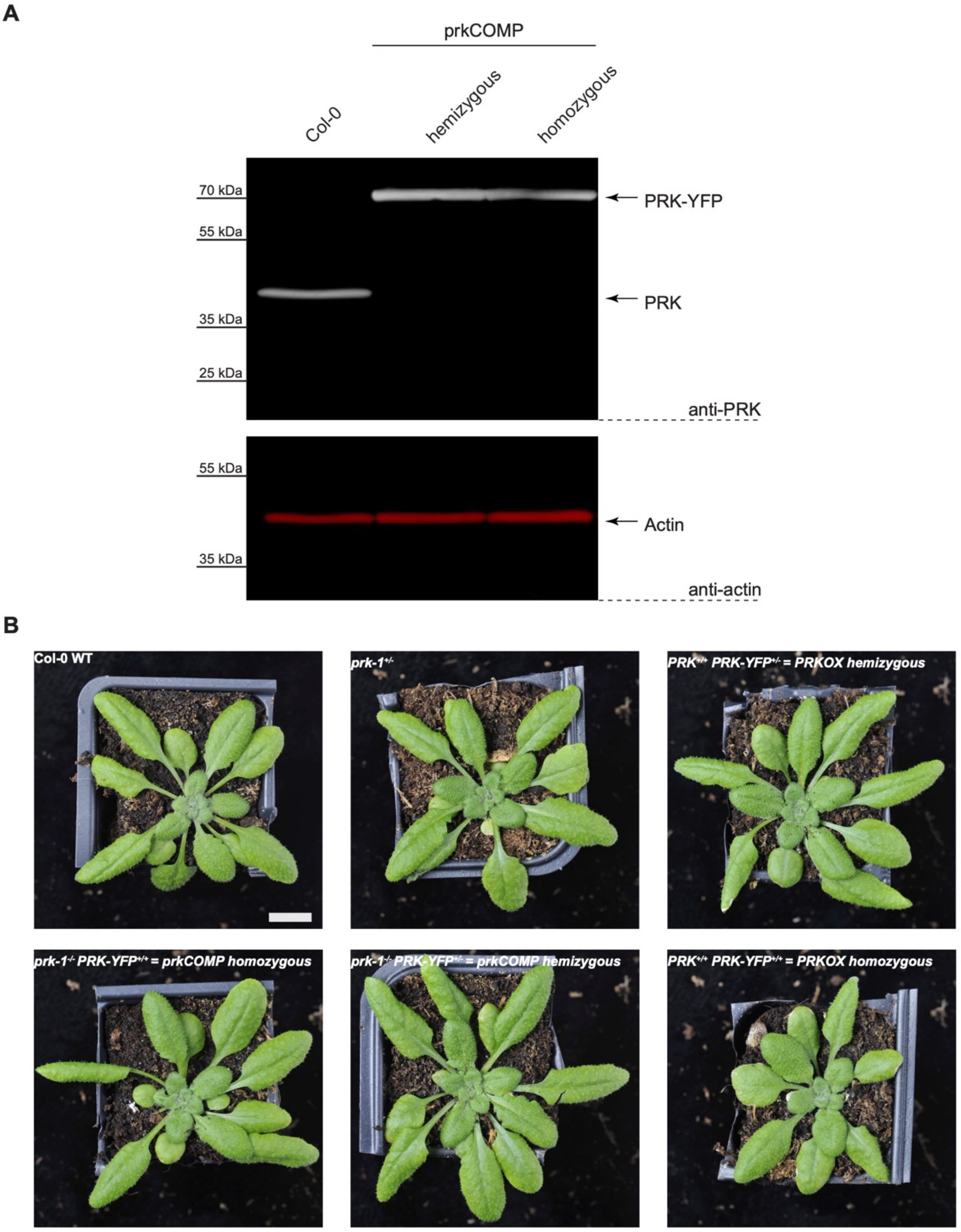

Supplemental Figure 1: Near-endogenous expression levels of PRK-YFP in one-month old prkCOMP rosette leaves, and prkCOMP/PRKOX growth (Supports Figures 2A and C).
A Immunoblot of protein extracts prepared from Col-0 WT and prkCOMP plants carrying either one or two copies of the *PRK$_{pro}$:PRK-YFP* rescue construct. Protein is loaded on equal fresh weight basis (500 ng each lane). **B** prkCOMP and PRKOX seeds originating from mother plants hemizygous for the *PRK$_{pro}$:PRK-YFP* construct were grown on soil under regular day-night cycles (12h light, 12h dark) for 5 weeks. Plants were genotyped to confirm *prk-1* background insertions and determine the zygosity of the *PRK$_{pro}$:PRK-YFP* construct. Scale bar, 1 cm.

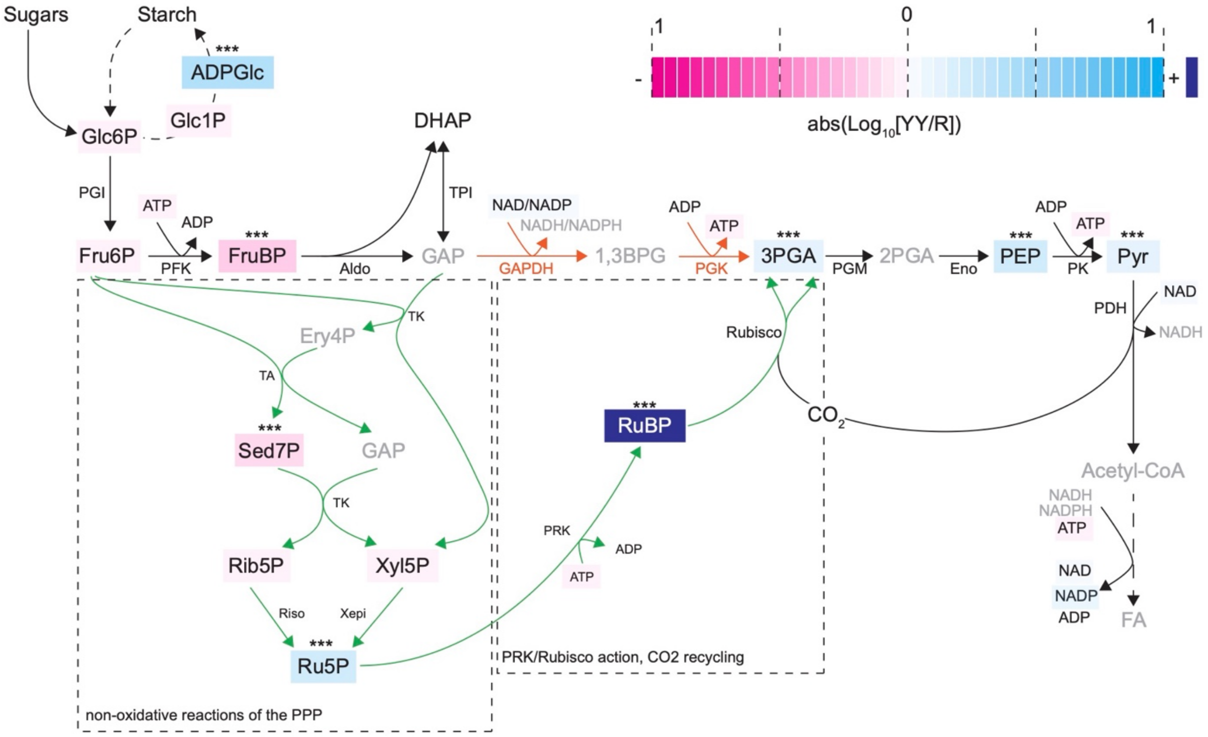

Supplemental Figure 2: The PRK/Rubisco shunt and changes in the involved metabolites upon overexpression of PRK in green embryos (Supports Figures 4 and 5A).
Same scheme as in Figure 4 (adapted from Schwender et al., 2004). Highlighted in color are relative changes in the mean metabolite levels in YY embryos with respect to R embryos originating from hemizygous PRKOX mother plants (log$_{10}$ scale; see color legend). RuBP was outside the displayed range, with a 35-times increase in YY samples. Statistically significant in- or decreases are indicated by asterisks; ***, $p < 0.001$ represents p values from 2-way ANOVA adjusted for multiple comparisons according to the Šidák method. Metabolites that were not analysed are displayed in grey font. See Supplemental Data Set 6A-C for Log$_{10}$-transformed metabolite values, a statistical analysis thereof, and relative changes, respectively.

Supplemental Table 1: Analysis of individual FA species in sorted prkCOMP embryos (data displayed in main Figure 5A as total FA). Shown are the means and SEMs of the FA species, expressed as a percentage of the total FA content, as well as statistical comparisons of the individual FA species between the prkCOMP, Y/YY and prkCOMP, R groups. Statistically significant differences, as based on a 2-way ANOVA with Sidak's multiple comparisons test (carried out in GraphPad Prism 9) are highlighted in blue and red (in- and decreased in R samples as opposed to Y/YY samples, respectively).

	prkCOMP, Y/YY			prkCOMP, R			Sidak's multiple comparisons test: prkCOMP, Y/YY – prkCOMP, R				
	Mean percentage	SEM of percentage	N	Mean	SEM	N	**Predicted (LS) mean diff.**	**95.00% CI of diff.**	**Below threshold?**	**Summary**	**Adjusted P Value**
16:0	7.224	0.071	23	6.922	0.062	24	0.3023	-0.1653 to 0.7700	No	ns	0.5913
16:1 (9Z)	0.158	0.031	23	0.092	0.021	24	0.06568	-0.4020 to 0.5333	No	ns	>0.9999
18:0	3.352	0.039	23	3.258	0.054	24	0.09429	-0.3734 to 0.5619	No	ns	>0.9999
18:1 (9Z) + 18.1 (11Z)	17.602	0.244	23	12.326	0.287	24	5.277	4.809 to 5.744	Yes	****	<0.0001
18:2 (9Z, 12Z)	29.042	0.135	23	25.508	0.208	24	3.534	3.066 to 4.002	Yes	****	<0.0001
18:3 (9Z, 12Z, 15Z)	18.21	0.196	23	24.38	0.294	24	-6.17	-6.638 to -5.702	Yes	****	<0.0001
20:0	1.83	0.03	23	2.132	0.048	24	-0.3018	-0.7695 to 0.1658	No	ns	0.594
20:1 (11Z) + 20:1 (13Z)	18.792	0.111	23	18.618	0.085	24	0.1746	-0.2931 to 0.6422	No	ns	0.9916
20:2 (11Z, 14Z)	1.693	0.023	23	2.796	0.048	24	-1.103	-1.571 to -0.6357	Yes	****	<0.0001
20:3 (11Z, 14Z, 17Z)	0.364	0.016	23	1.036	0.047	24	-0.6712	-1.139 to -0.2035	Yes	***	0.0004
22:0	0.196	0.01	23	0.274	0.015	24	-0.07845	-0.5461 to 0.3892	No	ns	>0.9999
22:1 (13Z)	1.424	0.022	23	2.278	0.051	24	-0.8545	-1.322 to -0.3868	Yes	****	<0.0001
22:2 (13Z, 15Z)	0.021	0.008	23	0.124	0.016	24	-0.1022	-0.5698 to 0.3655	No	ns	>0.9999
24:0	0.091	0.013	23	0.257	0.019	24	-0.1657	-0.6334 to 0.3019	No	ns	0.9951
24:1 (15Z)	0	0	23	0	0	24	0	-0.4676 to 0.4676	No	ns	>0.9999

Supplemental Table 2: Oligonucleotide primers used in this study.

Primer purpose	Primer 1 (5'-3')	Primer 2 (5'-3')
Genotyping: *prk-1*, wild-type band	CATACTCTGTTATGATACTCT	TGGAAAAGACCAGTACCGTTG
Genotyping: *prk-1*, T-DNA band	ATATTGACCATCATACTCATTGC	TTGTCATCGAAGGTCTTCACC
Genotyping: *prk-2*, wild-type band	CGAAGGTCTTCACCCAATGT	TTCGTTGTCATCTGGGATCA
Genotyping: *prk-2*, T-DNA band	CGAAGGTCTTCACCCAATGT	ACGGGATCCCGGTGAAACGGT
Cloning: *pPRK* promoter (attB4 and attB1r sites underlined)	GGGGGACAACTTTGTATAGAAAAGTTGTCTGCTTGGAGTCACTGCTG	GGGGGACTGCTTTTTTGTACAAACTTGTGTTGTTTGTTTGGTGTTTTGGTCT
Cloning: *PRK* CDS, lacking stop codon (attB1 and attB2 sites underlined)	GGGGACAAGTTTGTACAAAAAAGCAGGCTGGATGGCTGTCTCAACTATCTACTC	GGGGACCACTTTGTACAAGAAAGCTGGGTCGGCTTTAGCTTCTGCACGAGCAG
Genotyping: prkCOMP/PRKOX wild-type band	TGTTTGGATGAACTGATGAACAAAG	CTTCTAGTTTTATAGTTATGTAATC
Genotyping: prkCOMP/PRKOX insert	TGTTTGGATGAACTGATGAACAAAG	AACGTCCGCAATGTGTTATTAAGTTGTC
Cloning: *PRK-cTP* for recombinant expression (EcoRI and XhoI sites underlined)	TTTGAATTCATGGCACAAGAAACCATCGTG	AAACTCGAGGGCTTTAGCTTCTGCACGAGC
Cloning: *PRK-cTP* for recombinant expression, site-directed mutagenesis pair 1	CTAGCTGCTGACTCTGGCTCCGGCAAAAGT	ACTTTTGCCGGAGCCAGAGTCAGCAGCTAG
Cloning: *PRK-cTP* for recombinant expression, site-directed mutagenesis pair 2	GACACGACCACTGTGATCTCTCTTGATGATTACCAT	ATGGTAATCATCAAGAGAGATCACAGTGGTCGTGTC
