# Supplementary material for: The PRK/Rubisco shunt strongly influences Arabidopsis seed metabolism and oil accumulation, affecting more than carbon recycling
Source: Plant Cell. 2022 Dec 1;35(2):808–26. doi: 10.1093/plcell/koac338 (PMC9940875; doi:10.1093/plcell/koac338)
Supplement: koac338_Supplementary_Data [file tpc22.00670_PeerReviewReport.pdf › tpc22.00670_PeerReviewReport.pdf]

The PRK/Rubisco shunt strongly influences Arabidopsis seed metabolism and oil accumulation, affecting more than carbon recycling

Gabriel Deslandes-Hérold, Martina Zanella, Erik Solhaug, Michaela Fischer-Stettler, Mayank Sharma, Léo Buergy, Cornelia Herrfurth, Maite Colinas, Ivo Feussner, Melanie R. Abt, Samuel C. Zeeman

Corresponding author: *Samuel C. Zeeman (szeeman@ethz.ch)*

Review timeline:

**TPC2022-RA-00670**	Submission received:	July 13, 2022
	1st Decision:	August 26, 2022 *revision requested*
**TPC2022-RA-00670R1**	1st Revision received:	October 13, 2022
	2nd Decision:	Nov 1, 2022 *acceptance pending, sent to science editor*
	Final acceptance:	Nov. 18, 2022

REPORT: (The report shows the major requests for revision and author responses. Minor comments for revision and miscellaneous correspondence are not included. The original format may not be reflected in this compilation, but the reviewer comments and author responses are not edited, except to correct minor typographical or spelling errors that could be a source of ambiguity.)

TPC2022-RA-00670 1st Editorial decision – *revision requested* **August 26, 2022**

We have received reviews of your manuscript entitled "THE PRK/RUBISCO SHUNT STRONGLY INFLUENCES ARABIDOPSIS SEED METABOLISM AND OIL ACCUMULATION, AFFECTING MORE THAN CARBON RECYCLING." Thank you for submitting your best work to The Plant Cell. The editorial board agrees that the work you describe is substantive, falls within the scope of the journal, and may become acceptable for publication, pending revision and potential re-review.

We ask you to pay particular attention to the following points when preparing your revision:
1. Please provide a better justification for the use of only one complemented line. Multiple lines were generated, but it seems that only one line was characterized in detail.
2. Please clarify the significance of the reduction in lipid amounts. This part of your manuscript may benefit from a better explanation of the normalization method used. You seem to have normalized the lipid data on a per embryo basis, but what if embryo biomass is also impacted (see comment by Reviewer 1)?
3. The mutant embryos also show altered starch amounts. A more detailed discussion of how lipids and starch might influence each other would be appreciated. Could the change in lipid amount be a consequence of the change in starch or vice versa?
4. The analysis of flux by using metabolite levels as a proxy can be perilous, as flux may not correlate with metabolite levels. An appreciation of this point may ease the inconsistent interpretation of some pool size data that are observed in your manuscript.

-- Reviewer comments:

Reviewer comments shown below with author responses.

TPC2022-RA-00670R1 1st Revision received **October 13, 2022**

Reviewer comments and **author responses**:

Point 1. Please provide a better justification for the use of only one complemented line. Multiple lines were generated, but it seems that only one line was characterized in detail.

RESPONSE: We understand that phenotypic characterizations benefit from the comparison of several transgenic lines to reduce the risk of off-target and dosage-dependent effects of transgene expression. For the prk mutants, we characterized two independent alleles, which gave the same, largely predictable phenotype. For our prk complementation lines, however, we used a different strategy, obtaining in-depth information, well beyond the standard selection procedure for transgenic Arabidopsis lines, so as to be able to select the best one for detailed analyses. We 1) performed Illumina full genome sequencing to accurately map and characterise the transgene insertions, allowing us to select lines with single insertions in intergenic regions, 2) utilized the native promoter and confirmed near-endogenous transgene expression using a PRK-specific antibody, 3) established primers for genotyping so as to be able confirm zygosity at the determined transgene locus during every experiment, 4) used the selected line to produce the over-expressing PRKOX line, ensuring that the material is near-isogenic. Given the design of our stringent selection criteria, we feel we have minimized the risk of off-target effects, and that it was appropriate to focus our efforts on this best line. As shown, this line fully complemented the seedling-lethal prk-1 phenotype. We have added a more detailed description in the results section.

Point 2. Please clarify the significance of the reduction in lipid amounts. This part of your manuscript may benefit from a better explanation of the normalization method used. You seem to have normalized the lipid data on a per embryo basis, but what if embryo biomass is also impacted (see comment by Reviewer 1)?

RESPONSE: In all of our experiments, we did not discern a difference in seed size between those containing prk mutant embryos (R) and those containing rescued embryos (YY). To directly and accurately assess the weight differences in these different prkCOMP embryo classes at maturity would technically be very difficult, since their sorting at this stage is only possible after seed coat removal, necessitating seed imbibition. Having said that, from our data we would expect a weight difference. Arabidopsis Columbia seeds contain ~36% oil (Li et al., 2006). We therefore estimate that the 34% reduction in oil content seen in the case of prk embryos is indeed likely causing an embryo weight reduction - of maximally 12% (assuming that all other storage products/seed constituents are unaffected) and minimally ~6.5% (if the greater-than-expected reduction in oil is explained by channeling of carbon towards other seed components). Thus, since oil is a major seed component, and in the absence of changes in seed size, we feel that normalising to weight would actually be misleading. It would underestimate the impact of the prk mutation. We have elaborated on this in the discussion.

Point 3. The mutant embryos also show altered starch amounts. A more detailed discussion of how lipids and starch might influence each other would be appreciated. Could the change in lipid amount be a consequence of the change in starch or vice versa?

RESPONSE: Seed starch is dynamically turned over in Brassica seeds, which we tried to address by investigating it at different developmental stages of sorted prkCOMP embryos. Nevertheless, our current qualitative microscopy data and quantitative measurements do not have the temporal resolution to draw definitive conclusions, which we state in the revised manuscript. Having said that, our LCMS measurements provide important hints that starch synthesis is increased, at least transiently. While we have not measured the embryonic starch content at seed maturity, our imaging still shows, albeit qualitatively, that starch likely remains a minor component as compared to lipids. We have elaborated this point in the revised results and discussion sections, also taking into account the comments of Reviewer 2.

Point 4. The analysis of flux by using metabolite levels as a proxy can be perilous, as flux may not correlate with metabolite levels. An appreciation of this point may ease the inconsistent interpretation of some pool size data that are observed in your manuscript.

RESPONSE: We thank the reviewers and editor for raising this point, with which we agree 100%. Our study did not attempt to measure the metabolic fluxes directly, and our LCMS analyses were first and foremost done to confirm the anticipated changes in RuBP levels in the absence and upon overexpression of PRK. Nevertheless, the metabolite level data, together with the measurements of end-product accumulation, do allow us to speculate about changes in flux through the respective pathways. However, we have been more cautious in our interpretations and phrasing throughout the results and discussion.

Reviewer comments and **author responses**:

<u>Reviewer #1:</u>

During oil synthesis in developing oilseeds, maternally supplied sugar (sucrose) is converted into fatty acids and finally into triacylglycerols. In Brassicaceae, the RubisCO shunt has been described as a non-canonical sugar catabolic pathway that can increase the carbon efficiency of the conversion of sugars into fatty acids and acyl-lipids by recycling of CO2 produced by the pyruvate dehydrogenase complex. The shunt is bypassing two steps in glycolysis that produce ATP and NADH. The RubisCO shunt had been described in Brassica napus developing embryos based on isotope tracer and mass balancing experiments. Here, in-vivo relevance of the RubisCO shunt being tested by a genetic approach. *A. thaliana* has a single gene encoding for phosphoribulokinase (PRK), which produces the substrate RuBP for RubisCO. Plants depend on this gene for autotrophic growth (Calvin cycle) as well as the function of the RubisCO shunt. The authors analyzed two heterozygous PRK insertional mutant lines. Based on segregation analysis, homozygous seedlings were found to not be viable, which is to be expected since complete loss of PRK will not allow the Calvin cycle to take place. To analyze the effect of PRK loss on seed development, developing seeds of prk heterozygous lines need to be distinguished into their genotypes. To be able to distinguish homozygous mutant seeds from heterozygous and wild type seeds, the authors transformed heterozygous prk mutant plants with a construct expressing PRK merged with a fluorescent tag (YFP) under control of the endogenous PRK promoter. From this a line was selected that is hemizygous for the introduced transgene (pPRK:PRK-YFP), but homozygous for the prk background (line "prkCOMP"). The authors showed that segregating genotypes in prkCOMP can be distinguished at the seedling stage and during seed development. Developing seeds were sorted under a fluorescence microscope, allowing for biochemical analysis of genotypes R (chlorophyll fluorescence, non-complemented prk) as well as Y and YY seeds (YFP fluorescence, hemi- and homozygous transgenic seeds of pPRK:PRK-YFP). Metabolomic profiles of R, Y and YY green developing seeds isolated at 10 days after flowering were distinct from each other. RuBP, 3PGA, PEP and Pyruvate were found to be significantly reduced in R over YY seeds. ADP-glucose was found to be significantly increased. Total lipid content of embryos dissected from mature R seeds was 34% lower than that of Y/YY (weight per embryo). Besides FA accumulation, starch accumulation in developing seeds of R, Y and YY was affected as well. In wild type seeds, starch is transiently accumulated early in seed development, but almost completely disappears at maturity. Microscopic analysis of developing embryos showed that in R embryos the disappearance of starch during seed development was delayed. In addition to isolating lines expressing PRK-YFP in a PRK KO background (prkCOMP), the authors also isolated lines that express the PRK construct (pPRK:PRK-YFP) in the wild type background (line PRKOX), hereby creating an overexpression line. Relative to the wild type (fluorescence type R), PRKOX seeds had strongly elevated levels of the PRK reaction product RuBP. There were also increases in ADPGlc, 3PGA, PEP and Pyr in YY PRKOX seeds. However, lipid content per seed was not measurably increased. The authors take the strong accumulation of RuBP as an indication that upon increase in PRK activity, Rubisco activity may become limiting, which would explain why increased PRK would not significantly increase lipid levels.
The operation of the RubisCO shunt was also predicted to be dependent on contributions of ATP and NADPH from the photosynthetic light reactions in the developing seeds. Accordingly, shading experiments (shading of whole plant vs. shading only of the plant rosette, not siliques) showed that the light level has a positive impact on lipid levels in wild type seeds, but not in prk seeds.

Overall, the manuscript is very well written. With the key finding that a PRK KO causes a substantial reduction in fatty acid and lipid accumulation during seed development, this study adds an important piece of genetic evidence to the earlier studies that established the existence of the RubisCO shunt. Also, since the former studies were based on embryos cultured in vitro, this study is adding clear evidence of in-planta relevance of the pathway in A. thaliana developing seeds. The genetic evidence shown here was long overdue, but it is obvious that the genetic KO of PRK and the biochemical characterization of developing seeds devoid of RubisCO carboxylation required development of an elegant genetic strategy as was presented in the manuscript.

Point 1. With regards to the RubisCO shunt the authors refer to the original study that essentially established the pathway (Schwender et al., 2004)). For completeness, it might be worth mentioning additional studies that gave further support to the functioning of the RubisCO shunt in developing seeds, although all of them are studies applying in-vitro embryo cultures.
Lonien and Schwender Plant Physiol 151: 1617-1634 (2009): in embryos of A. thaliana (wild type), about 90% of 3PGA is generated by RubisCO.

Goffman et al. Plant Physiol 138: 2269-2279 (2005)
Schwender et al. Plant Physiol 168: 828-848 (2015): For different genotypes of Brassica napus, between about 40 and 70% of 3PGA is generated by RubisCO.
Tsogtbaatar et al. J Exp Bot 71: 3037-3051 (2020): In field pennycress developing embryos, about 25% of 3PGA is generated by RubisCO.

Also, an earlier study established the biochemical potential of developing seeds to re-fix CO2 at high rates. Ruuska et al. Plant Physiol 136: 2700-2709 (2004): "Rubisco was highly activated (carbamylated) in both light and dark, most likely reflecting high seed CO2 concentrations. Activities of Rubisco and phosphoribulokinase were sufficient to account for significant refixation of CO2 produced during B. napus oil biosynthesis."

> **RESPONSE: We are pleased that the reviewer appreciates our work and thank him/her for the complimentary and kind remarks on our manuscript. We further thank the reviewer for pointing out these important works and fully agree that they should be cited. We have integrated the references and their key findings into the present manuscript version, which has enhanced and brought better balance to the discussion.**

Reviewer #2:

This is an intriguing manuscript that investigates the role of Rubisco-based co2 fixation in oilseeds genetically through prk mutants. It builds on published work that described the non-canonical assimilation/re-assimilation of CO2 in oilseeds without a requirement for an intact Calvin cycle. The non-oxidative steps of the pentose phosphate pathway being supplied with hexose phosphate substrates from imported sugars are converted non-oxidatively to RuBP for Rubisco use. The Rubisco could reassimilate CO2 released in fatty acid biosynthesis of oilseeds, rendering the seeds more carbon efficient as has been previously hypothesized.

Point 1. Measure changes in seed number and size per plant (total biomass).

> **RESPONSE: We could not observe differences in the developmental progression of plants heterozygous for insertions in the endogenous PRK locus (e.g., heterozygous prk-1 or prk-2), or in our prkCOMP/PRKOX lines compared to the respective wild type, under the conditions we used. To convey this, we have re-grown these lines, genotyped them, and added representative pictures to the manuscript (See Supplemental Figure 1B).**

Point 2. Consider measuring ATP, ADP, AMP, NAD(P) and NAD(P)H to bolster any claims about their imbalance, particularly in the pale lines.

> **RESPONSE: We have measured levels of ATP, ADP, NAD, and NADP, (see main Figures 3 and 4, Supplemental Figure 2, and Supplemental Datasets 1 and 6) and did not observe statistically significant differences between the seed classes. On one hand, under conditions where cofactors are not efficiently utilized, we would expect a surplus. On the other hand, they may not be produced at the same rate in pale seeds. Thus, this part of the discussion remains speculative.**

Point 3. Did you provide data for more than one prk-COMP event? Some description of the COMP insert site is provided which helps minimize pleitropic considerations, though normally there is a view that at least two transgenic events should be preliminarily characterized with more depth on one line of interest, this would seem especially true for studies in a model, Arabidopsis?

> **RESPONSE: Please see comments to the editor and the corresponding changes to the manuscript's main text where we justify our approach.**

Point 4. If the TDNAs were hets is the presumption that 1 copy is mutated of 2 from the parents and there is only one PRK in Arabidopsis based on the genome? Probably this just needs to be clarified if that is the point.

> **RESPONSE: There is only one PRK gene in Arabidopsis. We isolated heterozygotes of two independent mutants and show that the homozygote mutants are seedling lethal. All subsequent work with the rescue construct was done either in a homozygous prk mutant background (viable thanks to the hemizygous rescue construct), or in the wild-type background. The offspring, resulting from self-fertilisation, are thus ¼ homozygous for the rescue T-DNA, ½ hemizygous (as the parental line) and ¼ lacking the rescue construct. However, they are all homozygous for either the mutant or wild-type PRK allele. We thank the reviewer for flagging up this potential misunderstanding and have double checked that we have used the right terminology throughout the manuscript.**

5. Lines 141-151 was confusing and I didn't understand UNTIL I read the legend for figure 1. Please state cotyledon stage of vegetative tissue growth, as there is a cotyledon stage also referencing reproductive development (heart, torpedo, cotyledon etc) which I assumed was the description initially. Even so, line 150 is confusing, because the pale homozygous lines arrested development at the cotyledon stage, yet there was no obvious effect on the developing seeds? But the development was arrested - i.e., no seeds were produced so how can you examine any effect? Maybe you mean the homozygous seed that was harvested and planted did not look different in appearance prior to planting?

RESPONSE: We thank the reviewer for pointing out this imprecise wording. We have further clarified this point in the manuscript's main text file.

Point 6. Why not partially characterize the leaves of the hets and pale homo line for net assimilation if you have access to a gas exchange instrument - this would be straightforward and help further make the case for the reduced Calvin cycle in mutants and presumably the pale lines have no net assimilation and are purely a consequence of metabolism of germination reserves.

RESPONSE: We agree with the reviewer's suggestion that the initial growth of the pale prk homozygous mutants is purely a consequence of metabolism of the seed reserves. Since these seedlings are very pale and arrest their growth at the cotyledon stage of vegetative development, it would be technically difficult to measure gas exchange rates. Gas exchange measurements on plants with different PRK amounts (e.g. the heterozygote, the wild type and the PRKOX lines we generated) would be interesting, but we feel this would be suitable for a follow up study. Since these plants all grow normally under the conditions we use (see the revised Supplemental Figure 1B), differences in gas exchange might only be apparent under certain conditions (e.g. saturating light).

Point 7. Why did the authors use high-light acclimated plants for the light study. The NL-Y/YY of high-light acclimated plants (Fig 5B) has a significantly reduced FA profile relative to normal light conditions for Y/YY (Fig 5A). Could the high-light acclimated plants have important physiological changes during the vegetative development. Did you consider a comparison of high-light (300 uE), medium light (200 uE) and low-light (100 uE) treatment when plants are grown in natural-light conditions from the seedling development. It would be helpful to report the % biomass basis and present it in supplements to see if it results in a similar conclusion.

RESPONSE: Based on earlier observations (Li et al, 2006), it was evident that even very minor differences in growth conditions that plants are experiencing can have a significant influence on final storage resource accumulation. To take this into account, we designed our experiments such that all external factors could be largely neglected. This included growing plants side-by-side under similar conditions, which would have been impossible when transferring individual mother plants to different light conditions. In addition, we reasoned that shifting low-light-acclimatized plants to high-light conditions could elicit stress responses, and the effects on lipid content may have been very difficult to dissect from such stress responses. In contrast, the converse, i.e. shifting high-light - acclimatized plants to low-light conditions, would be less stressful.

Point 8. Authors did not present the seed/embryo weight result of R and Y or YY samples. If the embryo weight in the R line is reduced, the lipid content as ug/embryo (Fig 5) can also be reduced even with similar fatty acid amount per mg of embryo biomass. On other hand, NO CHANGES in seed weight (if that is the case) would be also important when carbon efficiency should be reduced in prk mutant due to reduced CO2 refixation by Rubisco. It is important to analyze the different data based on % dry weight biomass.

RESPONSE: Please refer to our response to the editor and the corresponding changes to the manuscript main text file.

Point 9. I agree with the conclusion "starch is not properly mobilized during later development". However, "the biosynthesis of seed starch is upregulated in the absence of PRK" (line 295) is overstatement as authors could not find difference in the TEM result at earlier stage (7 and 13 DAF) when starch synthesis rate is generally higher.

RESPONSE: We feel that our TEM results at different stages are largely qualitative and we only carried out quantification of starch at one time point during embryo development. Thus, as detailed in our response to the editor, we can only speculate about differences in starch turnover in this context. Nevertheless, we should not ignore observations derived from our LC-MS data that point into the direction of increased starch synthesis, i.e. the increased ADPGlc and hexose phosphate contents of the R seed class from prkCOMP plants. We have reworded the

text so as to recognise the limitations of our data.

Point 10. 313: the logic for increased RuBP makes sense as a bottleneck and it may be the R5P accumulation is a further back up of metabolism, with Rubisco being the ultimate constriction. Its less clear that the PEP/PYR should be elevate per se if fatty acid production is not increased. Basically in the OX if PEP/PYR are elevated and FA is not changing because Rubisco is the pinch point than one would expect these levels to be the same as in WT. Line 443 makes a concession to this point in the discussion. 463 makes a good point about T3P being potentially limiting. Probably early in results where higher PYR/PEP is reported, the authors should be careful not to suggest it is a Rubisco issue when maybe it is more apt to be one of the others descriptions in discussion.

RESPONSE: It is difficult to interpret the increase in the Rubisco substrate RuBP in the PRKOX line as anything other than Rubisco activity being limiting, although we recognise (and now state in the manuscript) that this is surprising considering previously published data (e.g. Ruuska et al, 2004). We agree with the reviewer that the mildly increased levels of PEP and Pyr could be understood as either a flux enhancement through FA biosynthesis or stalling of the FA biosynthesis downstream, caused by other factors. However, as the editor pointed out, we have only carried out steady-state metabolic assessments and not flux studies. Thus, as described in the response to the editor, we have been more careful in our phrasing.

TPC2022-RA-00670R1 2nd Editorial decision – *acceptance pending* Nov. 1, 2022

We are pleased to inform you that your paper entitled "THE PRK/RUBISCO SHUNT STRONGLY INFLUENCES ARABIDOPSIS SEED METABOLISM AND OIL ACCUMULATION, AFFECTING MORE THAN CARBON RECYCLING" has been accepted for publication in The Plant Cell, pending a final editorial review by a science editor. At this stage, your manuscript will be evaluated by a science editor with respect to its presentation of scientific content, compliance with journal policies, and presentation for a broad readership.

Final acceptance from Science Editor Nov. 18, 2022
